# High-Resolution Small RNAs Landscape Provides Insights into Alkane Adaptation in the Marine Alkane-Degrader *Alcanivorax dieselolei* B-5

**DOI:** 10.3390/ijms232415995

**Published:** 2022-12-15

**Authors:** Guangshan Wei, Sujie Li, Sida Ye, Zining Wang, Kourosh Zarringhalam, Jianguo He, Wanpeng Wang, Zongze Shao

**Affiliations:** 1 School of Marine Sciences, Sun Yat-Sen University, Zhuhai 519082, China; 2 Key Laboratory of Marine Genetic Resources, Third Institute of Oceanography, Ministry of Natural Resources, Xiamen 361005, China; 3 State Key Laboratory Breeding Base of Marine Genetic Resources, Key Laboratory of Marine Genetic Resources of Fujian Province, Xiamen 361005, China; 4 Southern Marine Science and Engineering Guangdong Laboratory (Zhuhai), Zhuhai 519000, China; 5 Department of Mathematics, University of Massachusetts Boston, Boston, MA 02125, USA; 6 Center for Personalized Cancer Therapy, University of Massachusetts Boston, Boston, MA 02125, USA

**Keywords:** alkane, *Alcanivorax*, dRNA-seq, TSS, PSS, sRNA, metabolic regulation

## Abstract

Alkanes are widespread in the ocean, and *Alcanivorax* is one of the most ubiquitous alkane-degrading bacteria in the marine ecosystem. Small RNAs (sRNAs) are usually at the heart of regulatory pathways, but sRNA-mediated alkane metabolic adaptability still remains largely unknown due to the difficulties of identification. Here, differential RNA sequencing (dRNA-seq) modified with a size selection (~50-nt to 500-nt) strategy was used to generate high-resolution sRNAs profiling in the model species *Alcanivorax dieselolei* B-5 under alkane (*n*-hexadecane) and non-alkane (acetate) conditions. As a result, we identified 549 sRNA candidates at single-nucleotide resolution of 5′-ends, 63.4% of which are with transcription start sites (TSSs), and 36.6% of which are with processing sites (PSSs) at the 5′-ends. These sRNAs originate from almost any location in the genome, regardless of intragenic (65.8%), antisense (20.6%) and intergenic (6.2%) regions, and RNase E may function in the maturation of sRNAs. Most sRNAs locally distribute across the 15 reference genomes of *Alcanivorax*, and only 7.5% of sRNAs are broadly conserved in this genus. Expression responses to the alkane of several core conserved sRNAs, including 6S RNA, M1 RNA and tmRNA, indicate that they may participate in alkane metabolisms and result in more actively global transcription, RNA processing and stresses mitigation. Two novel CsrA-related sRNAs are identified, which may be involved in the translational activation of alkane metabolism-related genes by sequestering the global repressor CsrA. The relationships of sRNAs with the characterized genes of alkane sensing (*ompS*), chemotaxis (*mcp*, *cheR*, *cheW2*), transporting (*ompT1*, *ompT2*, *ompT3*) and hydroxylation (*alkB1*, *alkB2*, *almA*) were created based on the genome-wide predicted sRNA–mRNA interactions. Overall, the sRNA landscape lays the ground for uncovering cryptic regulations in critical marine bacterium, among which both the core and species-specific sRNAs are implicated in the alkane adaptive metabolisms.

## 1. Introduction

Alkanes, as a multifaceted issue, fuel human society but threaten ecological balance. In the ocean, alkanes are ubiquitous and can originate from both natural and anthropogenic activities, such as cyanobacterial biosynthesis [1], geothermal processes [2], seafloor seepages [3], and spills during the oil drilling and transporting [4]. *Alcanivorax*, as one of the most widespread and abundant hydrocarbon-degrading bacteria in the ocean, is particularly good at metabolizing various alkanes, regardless of aliphatic, branched, halo- and cyclo-alkanes [3,4,5,6,7]. Members of *Alcanivorax* play critical roles in the marine ecosystem through regulating the alkane cycling.

For decades, a number of studies have focused on the isolation, diversity and distribution of *Alcanivorax* based on the cultivation and culture-independent methods [4,8,9,10], and the key enzymes and pathways of alkane metabolism have been uncovered, especially in the *A. borkumensis* and *A. dieselolei* species [5,6,7,11,12]. Genes for multiple key enzymes usually coexist in their genomes, such as the AlkB, AlmA and cytochrome P450 alkane hydroxylases, to deal with different alkanes substrates [4,5,7,11,12,13]. In particular, the key genes in alkane sensing, chemotaxis, signal transduction, transporting and regulation have been revealed, and an entire network of alkane metabolism from sensing to degradation has been constructed in the model alkane-degrader *A. dieselolei* B-5 [7].

It has been well established that sRNAs are widely distributed both in various gram-positive and gram-negative bacteria, and they play diverse and widespread roles in almost all cell processes [14,15,16,17]. Bacterial sRNAs are at the heart of regulatory pathways, which allow bacteria to rapidly acclimate to the ever-changing environments through reshaping the related gene expression and adjusting their metabolism [14,15,18]. Generally, sRNAs can regulate gene expression through three different mechanisms: base-pairing to target RNAs (e.g., *trans*- and *cis*-acting sRNAs), directly interacting with proteins (e.g., 6S RNA and CsrA protein related sRNAs) and intrinsic functions (e.g., catalytic sRNAs) [17,18,19]. Given the highly dynamic environments and fluctuating alkanes content in the ocean [1,3], transient regulations via sRNAs are supposed to be essential to the quick adaptation of the ubiquitous and predominant alkane-degraders. However, knowledge of sRNA-mediated alkane metabolic regulations remains quite limited.

Bacterial sRNAs are usually regarded as the ‘dark matter’ in a genome due to their enormous diversity but difficulty to identify. Firstly, most sRNAs are poorly conserved except those within phylogenetically close species [20], resulting in the bad performance of bioinformatic predictions in most cases [14,21]. Secondly, the classical mutation-based genetic screening strategy to investigate protein-coding genes is usually inefficient for detecting sRNAs due to lacking of discernible phenotypes for most disrupted sRNAs [22,23]. Importantly, more and more evidence supports that sRNAs can originate from any location in the genome. In addition to the well-known intergenic and antisense regions, myriad sRNAs are derived from the UTRs (untranslated regions) and CDSs (coding sequences) of mRNAs [16,24,25,26,27,28,29,30]. Fortunately, with the rapid development of high-throughput sequencing technologies, RNA-seq-based approaches have been created to detect the global sRNA profiling [31], especially the differential RNA sequencing (dRNA-seq), which can even capture the sRNAs overlapped with other transcripts [32].

The dRNA-seq is an emerging technology for the global mapping of transcription boundaries and transcript origins with single-nucleotide resolution [33,34,35]. Owing to the unique advantage of differentiating transcription start site (TSS) and RNA processing site (PSS), dRNA-seq could detect sRNAs originating from any location in the genome [32]. Here, to identify the sRNA candidates in oil-degrading bacteria and infer the roles of sRNAs in alkane metabolism, dRNA-seq was used to capture the sRNA landscape in a model hydrocarbonclastic bacterium *A. dieselolei* B-5. Furthermore, we combined gene expression responses to alkane and targets analysis to predict the sRNA candidates and corresponding regulatory mechanisms in alkane metabolisms. The results provide insights into sRNA-mediated alkane adaptability of the prevalent marine alkane-degrader.

## 2. Results and Discussion

### 2.1. dRNA-seq Uncovering Genome-Wide sRNA Profiles with High-Resolution of 5′-Ends

To verify the accuracy of 5′-end of transcripts in dRNA-seq, we first compared the start sites of highly conserved tRNAs predicted by two independent methods (tRNAscan-SE 2.0 and ARAGORN) [36,37] with the results annotated by dRNA-seq (Table S1). As a result, the vast majority of tRNAs (42/49) were predicted exactly at the same start sites by the two methods, in which most of them (39/42) have a precisely mapped TSS or PSS in the dRNA-seq results (Table S1). The relatively small tRNAs (with sizes of ~70-nt to 100-nt), as well as the larger conserved tm-RNA (358-nt) predicted by Bakta software tool [38], showed the same start site with the dRNA-seq results (Table S1). The few other unmapped tRNAs might be caused by the incorrect prediction or silenced expression in the detected conditions. Moreover, the relative expression levels of the matched tRNAs and tm-RNA spanned a great range of six orders of magnitude (with TPM from 1 to 10^6^), indicating that both rare and abundant transcripts are detected. Therefore, the dRNA-seq could capture the 5′-end of transcripts with different sizes and varied expression levels at the single-nucleotide resolution in this study.

**Figure 1:**
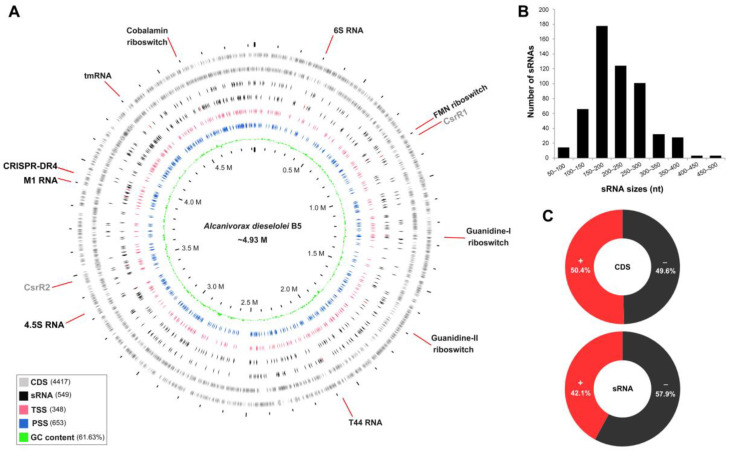
Genome-wide sRNAs landscape of A. dieselolei B-5. (**A**) Diagram shows the distributions of sRNAs, TSSs and PSSs across the B-5 whole genome. From outside to inside, the rings represent the distributions of CDSs, sRNAs, TSSs, PSSs and the GC content, respectively, and the values in brackets show corresponding numbers or percentages in the genome. The orientations of each ring are clockwise for the plus (+) strand and counterclockwise for the minus (−) strand. The relatively conserved sRNAs are red marked on the corresponding rings, and their annotated names are displayed on the outermost edges of the plot; black fonts for Rfam families and grey for identified CsrA-related sRNAs. Circos plot was created by using Proksee (https://proksee.ca/, accessed on 23 March 2022). (**B**) The size distribution of sRNAs. (**C**) Comparison of the distributing proportions of CDSs and sRNAs in two strands (+/−) of the genome.

To gain comprehensive profiles of the sRNAs, we combined the ANNOgesic prediction and manual inspection based on the dRNA-seq and ssRNA-seq data [32,39]. Finally, a total of 549 sRNA candidates were obtained in strain B-5 under both alkane and non-alkane conditions (Figure 1A and Table S2). Given the accurate and unique 5′-end locations on the genome, the sRNAs are named in the form of sRNAXXX, of which XXX stands for the corresponding specific genomic location of each sRNA (Table S2). Generally, most of the sRNAs (~85%) ranged from 100-nt to 300-nt in length, with a median of 205-nt (Figure 1B). For the strand distribution, sRNAs showed more minus-strand preference than CDSs in the genome (Figure 1C). Among these sRNAs, 2.6% of them (14/549) showed high similarities (>70%) with at least one other sRNA detected in this study, indicating that few sRNAs are redundantly expressed in B-5 genome. Interestingly, nearly half of these redundant sRNAs (6/14) are antisense transcripts of different transposase genes in the B-5 genome (Table S3). This suggests that the transposition activities of transposons may be widely repressed at a post-transcriptional level through antisense sRNAs in the B-5 genome, which is also reported in other bacterial and archaeal species before [40].

### 2.2. Origin Patterns of sRNAs Associated with Promoters and RNase E Cleavages

**Figure 2:**
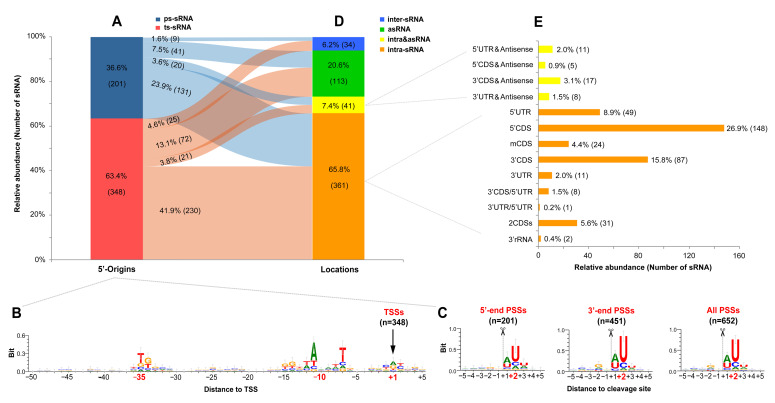
Origin and location patterns of sRNAs. (**A**) Distribution of sRNAs classified by the origins of 5′-ends. (**B**,**C**) show the sequence logos of neighboring regions of TSSs and PSSs, respectively. The “−” and “+” before the numbers represent the upstream and downstream positions, respectively, and the featured positions are shown with bold and red fonts. (**D**,**E**) show the distributions of sRNAs classified by their locations relative to the genomic annotations. The percentage represents the ratio of one type of sRNA in the total, and the following number in the bracket shows the corresponding amount. The relationships between (**A**) and (**D**) are connected by a Sankey diagram created by using the SankeyMATIC (https://sankeymatic.com/build/, accessed on 28 February 2022).

To understand the origin patterns of sRNAs in the genome, considering the accurate TSSs and PSSs obtained by dRNA-seq, we first classified the sRNAs based on their 5′-end origins into two types: ts-sRNA (transcription starting sRNA) and ps-sRNA (processing sRNA) (Figure 2A and Table S4). The ts-sRNAs originated from their own transcription start sites and account for approximately two-thirds of the total sRNAs, which are more abundant than ps-sRNAs derived from the processing of other transcripts (Figure 2A). For the 348 TSSs, the sequence logo of the upstream 50-nt displayed typical motif characteristics of bacterial promoters in the −10 and −35 regions (Figure 2B). Meanwhile, iPromoter-2L-based prediction indicated that about half of the upstream regions of TSSs could be recognized by distinct sigma factors, especially by σ70 (40.2%) and σ24 (12.4%) (Table S5). Furthermore, an obvious A/G purine preference at the TSS (+1 position) was also detected (Figure 2B), which is well consistent with previous reports from other bacterial transcription start sites [41,42].

For the PSSs at the 5′- and 3′-ends of sRNAs, the sequence logos of their neighboring sequences showed a strong preference for uridine (U) at the downstream 2-nt (+2 position) of cleavage sites, and a 5-nt consensus motif of “RN↓WUU” (with “↓” for “cleavage site”, “R” for “G/A”, “W” for “A/U”, and “N” for “any nucleotide”) was identified (Figure 2C and Table S6), reminiscent of the essential RNase E in Gram-negative bacteria, which prefers to cleave RNA in single-stranded A/U-rich regions [43,44]. Unexpectedly, the motif feature of *A. dieselolei* matches the RNase E-specific recognition motifs in diverse bacteria very well (Figure S1), including *Salmonella enterica* [45], *Vibrio cholerae* [27] and even the model cyanobacterium *Synechocystis* sp. PCC 6803 [46]. This highlighted that RNase E should be the overriding player in the processing of sRNAs in *A. dieselolei*, and the inference is further supported by the highly conserved crucial residues for specific recognition and cleavage of RNase E between *A. dieselolei* and the aforementioned bacteria (Figure S1). These results indicate that the origin patterns of ts-sRNAs and ps-sRNAs are closely associated with upstream promoters and RNase E cleavage sites, respectively.

### 2.3. Diverse Location Patterns Relative to the Annotated Genes in the B-5 Genome

To understand the sources of the sRNAs in the B-5 genome, they were also classified based on their locations relative to the annotated genes. As a result, the sRNAs were distributed in diverse genomic locations and can be divided into three major types: inter-sRNA (intergenic sRNA), asRNA (antisense sRNA) and intra-sRNA (intragenic sRNA) (Figure 2D and Table S4). The inter-sRNA is located on the intergenic region of two nearby genes and without overlap with transcripts of the two genes; the asRNA overlaps with the transcript of annotated gene, but with a divergent transcriptional direction; the intra-sRNA overlaps with the partial transcript of the annotated gene with same transcriptional direction (see detailed examples in Figure S2). According to the specific locations of the overlaps, the intra-sRNAs can be further subdivided into sRNAs in 5′UTR (at the 5′UTR of mRNA), 5′CDS (overlapped with the 5′-end of CDS), mCDS (at the middle of CDS), 3′CDS (overlapped with the 3′-end of CDS), 3′UTR (at the 3′UTR of mRNA), 3′rRNA (overlapped with the 3′-end of rRNA), 2CDSs (overlapped with two same directional CDSs) and so on (Figure 2E and Figure S2 and Table S4). In addition, some other sRNAs that overlap with two transcripts of nearby genes in divergent directions are designated as intra&asRNA (Figure 2D,E and Figure S2). The majority of our retrieved sRNAs belonged to intra-sRNAs (65.8%), particularly sRNAs overlapped with the 5′- and 3′-ends of mRNAs (Figure 2D,E), indicating that mRNA may be the primary origination source of sRNAs in *A. dieselolei*. This finding is in congruence with recent evidence in different bacteria, such as sRNAs from 3′UTRs in *Salmonella enterica* [45], 5′UTRs and CDSs in *Escherichia coli* [24,26], and CDSs, 5′UTRs and 3′UTRs in *Xanthomonas campestris* [30].

### 2.4. Sequence and Structure Features of sRNAs Associated with the Location Patterns

**Figure 3:**
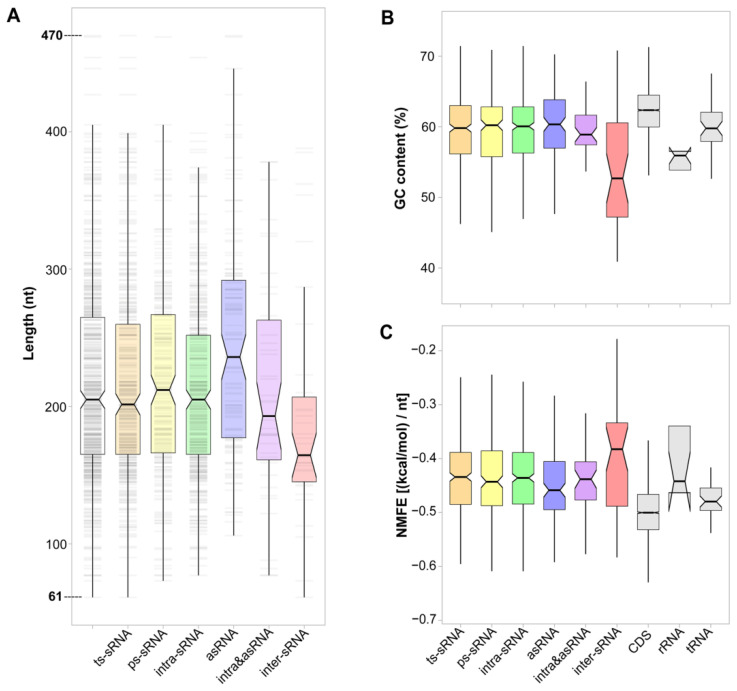
Sequence and structure features of sRNAs with different classifications. (**A**) Boxplot shows the length distributions of different types of sRNAs. The grey stripes in the background shows the overall length distributions. (**B**) GC contents and (**C**) NMFEs of classified sRNAs, CDSs, rRNAs and tRNAs.

The sRNA features, including sequence length, GC content and minimum free energy were calculated and compared according to the above classifications. The sequence lengths of all sRNAs ranged from 61-nt to 470-nt, with an average of 217 ± 72-nt (Figure 3A and Table S7). For different types of sRNAs, ts-sRNA (217 ± 72-nt) and ps-sRNA (217 ± 71-nt) showed no significant difference in length (Wilcoxon rank sum test, *p* > 0.05), while the asRNAs (244 ± 81-nt) and inter-sRNAs (193 ± 84-nt) had notably longer and shorter sizes than others, respectively (*p* < 0.05, Table S7). In a comparative investigation based on 816 identified sRNAs across 33 bacterial species, the authors summarized that the average lengths of *cis*- and *trans*-sRNAs are 219- and 182-nt, respectively [21], which is very similar to the length of the distributions of corresponding asRNA and inter-sRNA in our study.

The inter-sRNAs have the lowest GC content (53.9% for average), which is comparable to rRNAs (55.4%) but significantly lower than other types of RNAs (58.4–61.8%). Except for the inter-sRNA, all other types of sRNAs had similar GC contents (58.4–59.8%), which are significantly lower than CDSs (61.8%), higher than rRNAs (55.4%) and comparable with tRNAs (60.1%) (Figure 3B and Table S7). However, both intergenic and antisense sRNAs usually had relatively lower GC contents than the CDSs, rRNAs and tRNAs in other bacterial species [21,47]. Of note, most of the previously reported sRNAs are usually derived from the genomes of human gut bacteria, such as *Escherichia*, *Salmonella* and *Bacteroides*, which have considerably lower GC contents (~54% for average) than our marine-derived strain B-5 (61.6%) [21,47]. Although the GC content has been used as predictive information for intergenic sRNA identification in some bacteria [48], our results indicated that the GC contents of sRNAs vary in different genomic locations and distinct species.

The secondary structure feature of sRNA is reflected by the length normalized minimum free energy (NMFE), which is closely correlated with the structural folding stability of RNA [21]. Except between inter-sRNA (−0.3993 (kcal/mol)/nt for average) and asRNA (−0.4478), all the different types of sRNAs showed no significant difference in NMFEs. However, all the sRNAs showed remarkably higher NMFEs (ranging from −0.3993 to −0.4478) than that of the CDSs (−0.4955) and tRNAs (−0.4800), and were comparable with the rRNAs (−0.4148) (Figure 3C and Table S7). The higher NMFEs of sRNAs may correlate with their less stable secondary structures, since the sRNA-chaperone Hfq protein is also a key factor correlated with their stability through protecting sRNAs from degradation [21,49,50]. Furthermore, the NMFEs of different types of RNAs showed a strongly negative correlation with their average GC contents (Spearman’s ρ = −0.95, *p* < 0.001) in this study (Table S7), indicating the close relationship between RNA structure stability and GC contents.

Taken together, the sequence- and structure-based features of sRNAs are less influenced by their origin patterns (i.e., transcription starting or processing origins), but are closely correlated with their location patterns (i.e., intergenic, intragenic or antisense) in *A. dieselolei*. It is noted that these features may provide some valuable parameters for the bioinformatic prediction of sRNAs in the genome, and the differences caused by the species-dependent specificity should be fully considered.

### 2.5. Protein-Coding Potential of sRNAs

The ORF Finder prediction showed that about one-third of the asRNAs (35/113) or inter-sRNAs (12/34) have coding potential with at least one small ORF (Table S8). Ribo-seq result showed that only 7.3% sRNAs (40/549) contain typical small ORFs (sORFs) (Table S9), and this proportion is similar with a previous report on several bacterial phyla (5–15%) by a machine learning prediction method [51]. BLASTP results further showed that most of the putative coding sRNAs were either hypothetical proteins or without known homolog in the NCBI nr database (Table S9). Intriguingly, an additional 136 sRNAs even without coding potential were also detected in the Ribo-seq data (Table S9). A similar phenomenon was also observed in the Ribo-seq results of *E. coli*, and the persistent existence of sRNAs was attributed to their protective secondary structures instead of the ribosome binding [52]. Another possible explanation is that most of the 136 sRNAs belong to the intra-sRNAs, in which the ribosomes may simultaneously protect these sRNAs when binding their parental mRNAs. Therefore, both the bioinformatic prediction and the Ribo-seq results support that most of the sRNAs are still non-coding in this study. Considering the difficulty of sORF validation [51], the limitation of Ribo-seq [52] and the bifunctional (both sORF-coding and base-pairing regualtion) potential [53], these sRNAs with coding potential were not excluded from the next regulatory analyses.

### 2.6. Identification of Rfam-Annotated Core sRNAs

**Figure 4:**
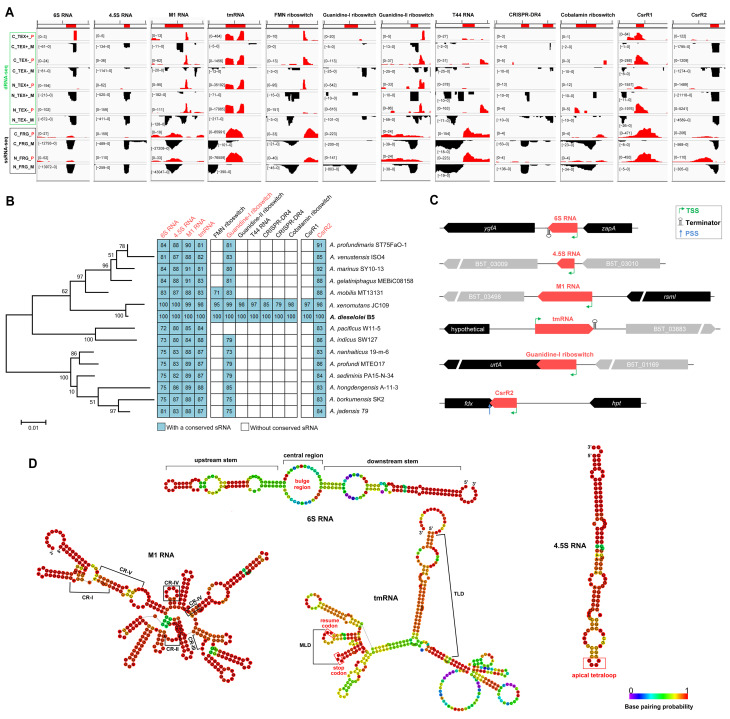
Rfam-annotated sRNAs of *A. dieselolei* and their distributions in genus *Alcanivorax*. (**A**) IGV views on the read coverages of Rfam-annotated sRNAs. Tracks display the different experimental conditions (i.e., C for C16 (hexadecane) and N for NaAc (sodium acetate) carbon sources) and RNA-seq strategies (i.e., TEX+ and TEX- for dRNA-seq and FRG for ssRNA-seq). The reads mapped on the plus (P) and minus (M) strands of the genome are in red and black colours, respectively. (**B**) The homologs of Rfam-annotated sRNAs in Alcanivorax. The left part shows the 16S rRNA gene-based maximum-likelihood phylogenic tree of representative *Alcanivorax* species constructed using MEGA6 (www.megasoftware.net, accessed on 19 May 2022), the bootstrap values that are more than 50 are presented on the related branches. The middle part shows the distributions and identities (%) of sRNAs in different species, and the names of broadly conserved sRNAs are in red. The right part shows the names of corresponding species. (**C**) Neighboring gene synteny of highly conserved sRNAs. The red arrows in the middle represent the sRNAs, black for the highly conserved nearby genes and grey for the less conserved genes. The arrow direction to the left indicates genes on the minus-strand and to the right for the plus-strand. The details of the nearby genes are listed in Table S12. (**D**) Secondary structures and conserved features of the core sRNAs.

To reveal the sRNAs with homologs in the database of known RNA families, all sRNA candidates were aligned in the Rfam database. As a result, 11 of the 549 sRNAs were successfully annotated into 10 Rfam families, including a 6S RNA, a 4.5S RNA, a M1 RNA, a tmRNA, a T44 RNA, two CRISPR RNA direct repeat elements (CRISPR-DR4) and four different riboswitches (Figure 1A and Figure 4A and Table S10). All of these sRNAs were detected in both the dRNA-seq and ssRNA-seq results with clear read peaks (Figure 4A), and they mainly originate from the intergenic or 5′UTR regions with their own TSSs (Table S10). BLASTN similarity searches against the 15 reference genomes of *Alcanivorax* suggested that only 4 of them, i.e., 6S RNA, 4.5S RNA, M1 RNA and tmRNA, are widely distributed in the *Alcanivorax* genus (Figure 4B). In fact, these four sRNAs are also broadly distributed in other bacteria and play key roles as core sRNAs, and their detailed features in *Alcanivorax* are as follows.

#### 2.6.1. 6S RNA and Associated pRNAs

The 6S RNA is an important global riboregulator at the transcriptional level through mimicking the open DNA promoter to sequester the RNA polymerase (RNAP) in *Escherichia coli* and *Bacillus subtilis* [17]. Since the conserved primary sequence is lacking, it is usually hard to predict the 6S RNA gene in other non-model bacteria [39]. Here, through combined dRNA-seq and Rfam alignment, a 197-nt 6S RNA was first identified from *A. dieselolei* and is conserved in *Alcanivorax* with varied sequence similarities (Figure 4B). Gene synteny and Rfam annotation of all the homologs further confirmed the conservation of 6S RNA in *Alcanivorax* (Figure 4C and Tables S11 and S12). The upstream neighboring gene is the *zapA* encoding cell division protein, and the downstream gene *ygfA* encoding 5-formyltetrahydrofolate cyclo-ligase is involved in purine metabolism (Figure 4C and Table S12). The *zapA-6S RNA-ygfA* synteny is also reported in other bacterial taxa, especially the *Gammaproteobacteria* [54], implying some underlying relationships between 6S RNA and cell division and nucleic acid metabolism. Further secondary structure prediction indicated that *A. dieselolei* 6S RNA displays a structure of “upstream stem—central bulge—downstream stem” (Figure 4D), which is in line with the typical structure of *E. coli* 6S RNA [55]. Importantly, we obtained evidence for the existence of 6S RNA-associated product RNAs (pRNAs) nearby the central bulge region (Figure S3), which may mediate the release of sequestered RNAP by 6S RNA [17]. In fact, four putative pRNAs with different 5′-ends and varied lengths were uncovered in *A. dieselolei* (Figure S3), which are more diverse than previously described, such as in *Helicobacter pylori* and *Bacteroides thetaiotaomicron* (two potential pRNAs), in *E. coli* and *B. subtilis* (one pRNA), and in *Legionella pneumophila* (without pRNA) [17,34,47]. The diverse pRNAs may contribute to the flexible release of the sequestered RNAP by 6S RNA to rapidly recover the transcriptional activity globally, which is critical to the survival of *Alcanivorax* in the ever-changing marine environments.

#### 2.6.2. 4.5S RNA of SRP

The bacterial signal recognition particle (SRP) RNA, termed 4.5S RNA in *E. coli*, is the core component of SRP for delivering inner membrane proteins to the translocon or the insertase for membrane insertion [39,56]. The 113-nt 4.5S RNA of *A. dieselolei* showed 80–88% sequence identities with that in most of the *Alcanivorax* species (Figure 4B), and all homologs were confirmed in the Rfam database (Table S11). However, the neighboring gene synteny of 4.5S RNA is not conserved among *Alcanivorax* species (Figure 4C and Table S12). The secondary structure of 4.5S RNA forms a hairpin-like structure with several internal loops and a conserved apical GGAA tetraloop (Figure 4D), which well resembles that of *E. coli* [56].

#### 2.6.3. M1 RNA of RNase P

M1 RNA is the core catalytic component of RNase P in bacteria that participates in the processing and maturation of various RNAs, like tRNAs, tmRNA, 4.5S RNA, and some mRNAs [19,57,58]. We obtained a 362-nt M1 RNA from *A. dieselolei*, which has widespread homologs with high similarities in *Alcanivorax* representative strains (Figure 4B and Table S11). A highly conserved *rsmI* gene encoding 16S rRNA (cytidine (1402)-2’-O)-methyltransferase is located upstream of the M1 RNA gene, but the downstream genes are not conserved in *Alcanivorax* (Figure 4C and Table S12). Moreover, the five universally conserved regions (CR-I to CR-V) and key catalytic sites of M1 RNA were also identified in *Alcanivorax* (Figure 4D and Figure S4), further confirming its functional conservation as the core component of RNase P resembling other identified bacteria [57,59].

#### 2.6.4. tmRNA and Its Flexible MLD

Another core sRNA from *A. dieselolei* is a 388-nt transfer-messenger RNA (tmRNA), which plays a vital role in rescuing the stalled ribosomes on defective mRNAs in bacteria [60]. The *Alcanivorax* tmRNAs shared a conserved upstream gene encoding a hypothetical protein in an opposite transcriptional direction (Figure 4C and Figure S5). Like in *E. coli*, a key mRNA-like domain (MLD) was also identified in *A. dieselolei* tmRNA (Figure 4D), which features an internal ORF encoding a small protein (10 amino acids) to tag the problematic peptides produced by the stalled ribosomes for protease recognition [60]. Interestingly, the resume codon (GCA) and stop codon (UAA) of MLD are highly conserved in *E. coli* and *Alcanivorax* species (Figure 4D), but the fourth and the fifth codons encoded different amino acids with similar physicochemical properties (Glu-Asn for *E. coli* and Asp-Thr/Ser for *Alcanivorax*) (Figure S5). Moreover, nearly half of the MLD codons of *Alcanivorax* showed base wobbles mainly in the third position, but most of the corresponding amino acids are still unchanged (Figure S5). Different bacterial species use distinct codons to encode flexible protein-tags in MLD of tmRNA, which probably depends on their codon preferences, and the functions of the protein-tags may remain constant due to the degeneracy of codon and similar properties of amino acids.

### 2.7. Identification of Rfam-Annotated Locally Distributed sRNAs

In addition to the above four core sRNAs, some Rfam-annotated locally distributed sRNAs that are conserved in some bacteria are also identified from *A. dieselolei*.

#### 2.7.1. Riboswitches Related to Detoxication of Guanidine and Metabolism of B Vitamins

Riboswitch is one of the most common riboregulators in bacteria, which recognizes the specific small molecular ligand by its aptamer domain and modulates the downstream gene transcription or translation through switching the conformation [61]. Guanidine-I riboswitch is widely distributed in most species of *Alcanivorax* and conservatively located at the upstream of an *urtA* gene encoding a putative urea ABC transporter substrate-binding protein (Figure 4B,C). The Guanidine-II riboswitch is only detected in *A. dieselolei* and its closest species *A. xenomutans*, with a downstream gene encoding a SugE-like protein (Figure 4B and Table S10). Guanidine is derived from the metabolisms of arginine, creatine or guanine, which is a toxic compound to the cell [62]. Guanidine riboswitches play crucial roles in the degradation or export of this toxin through regulating expressions of the downstream guanidine carboxylase or transporter genes [62]. Considering the downstream genes and distribution patterns, most *Alcanivorax* members may be capable of exporting the intracellular guanidine via the sensing and regulation of Guanidine-I riboswitch, and *A. dieselolei* and *A. xenomutans* may have more powerful guanidyl-scavenging abilities due to their extra Guanidine-II riboswitch.

The other two riboswitches are closely related to the metabolism of B vitamins, with FMN riboswitch and Cobalamin riboswitch for the regulation of biosynthesis or transport of riboflavin (vitamin B2) and cobalamin (vitamin B12), respectively [63,64]. Although Cobalamin riboswitch and FMN riboswitch are among the top three most widely distributed riboswitches in bacteria [61,63], they showed limited distribution in *Alcanivorax* (Figure 4B). Based on the functions of downstream genes (Table S10), we deduce that *A. dieselolei* and the closely relatives may modulate their intracellular vitamin B2 and B12 concentrations through FMN- and Cobalamin-riboswitch-mediated riboflavin biosynthesis and cobalamin uptake, respectively. Note that despite most of the known riboswitches controlling gene expression through a *cis*-acting way [61], the above four riboswitches in *Alcanivorax* showed clearly accumulated transcription peaks (Figure 4A), and two of them have PSSs at their 3′-ends (Table S10), indicating their *trans*-acting regulation potential as sRNAs, which is also reported in other bacteria [39,65,66].

#### 2.7.2. T44 RNA Likely Associated with Factors of Translation Process

T44 RNA was first reported in *E. coli* in a microarray-based transcriptome study about twenty years ago [67], and it was found to be widely distributed in different bacteria, but with unknown functions ever since [68]. Here, we also identified a T44 RNA from *A. dieselolei*, located between the genes *map* (encoding methionine aminopeptidase involved in the amino-terminal maturation of translation process) and *rpsB* (encoding 30S ribosomal protein S2) (Figure 4A and Table S10), which is consistent with most bacteria [68]. Intriguingly, based on the dRNA-seq results, T44 RNA is antisense and overlapped to the 5′UTRs of *map* and *rpsB* genes, respectively (Figure S6). Meanwhile, T44 RNA probably shared the same TSS with the two downstream genes *rpsB* and *tsf* (encoding translation elongation factor Ts) (Figure S6), which is similar to the *Vibrio splendidus* [68]. Given that of all the related *rpsB*, *tsf* and *map* genes are involved in the translation processes, we propose that T44 RNA may influence global translation through the *cis*-acting effects on these genes in *A. dieselolei*.

#### 2.7.3. Two Active CRISPR RNAs within a Type I-F CRISPR-Cas System

In *A. dieselolei*, we also identified two species-specific CRISPR RNAs (crRNAs) of the CRISPR-DR4 family, which is characterized by the same repeat with palindrome to form a 6-bp stem-loop with high GC contents (Figure 4A,B and Figure S7). The CRISPR-Cas system is the adaptive immune system in most prokaryotes against the phages as well as mobile genetic elements [69]. Based on the CRISPRCasFinder [70], a Type I-F CRISPR-Cas system was identified in the *A. dieselolei* genome, and both detected crRNAs are within a large CRISPR array containing 63 distinct spacers (Figure S7). The 159-nt crRNA sRNA3935639 originated from a TSS and contains three different spacers separated by two repeats, while the 209-nt sRNA3936114 is derived from a PSS and has four spacers separated by three repeats (Table S10 and Figure S7). Compared to the common mature crRNA that usually has one spacer–repeat pair, here the two crRNAs with multiple spacer–repeat pairs indicate a complex processing mechanism of the CRISPR array with larger stable intermediates, which is also seen in type I-B systems of bacterial *Fusobacterium nucleatum*, *Clostridium thermocellum* and archaeal *Methanococcus maripaludis* [39,71]. Interestingly, both of the active crRNAs contain one spacer that has a putative seed sequence targeting the integrated phage genes with 14-nt perfect matches in the *A. dieselolei* genome (Figure S7), and both the targeted genes belong to tailed and double-stranded DNA bacteriophages of the order *Caudovirales* (Table S13).

### 2.8. Distribution Characteristics of sRNA Homologs

**Figure 5:**
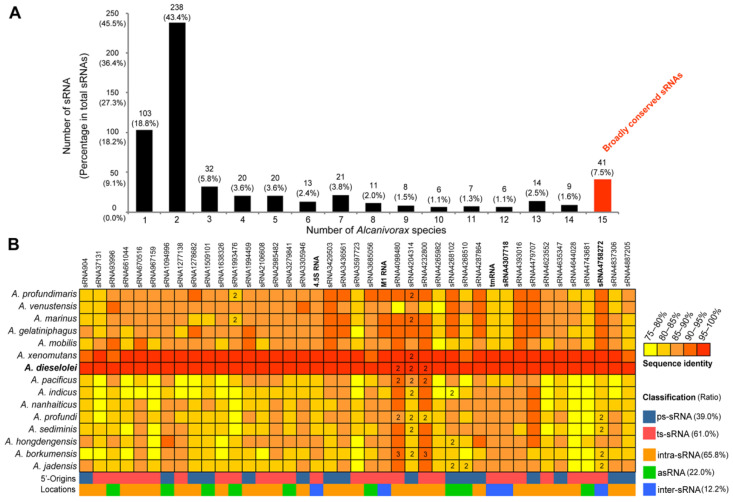
Distribution of sRNA homologs across different species of *Alcanivorax*. (**A**) The number (percentage) distribution of sRNA homologs across one (*A. dieselole*i-specific) to all 15 (broadly conserved) species of *Alcanivorax*. The bar with broadly conserved sRNAs is marked in red. (**B**) The sequence identity distribution of the 41 conserved sRNAs across different *Alcanivorax* species and their classifications in the reference strain B-5. The different sequence identities are shown using color gradients from yellow to red. The number (2 or 3) in the grid represents the copy number of the related sRNA in corresponding species, and the average identity of multiple copies is shown. The classifications of sRNAs are also displayed by distinct colors.

Except for a few Rfam-annotated sRNAs, most of the sRNAs (538/549) detected in *A. dieselolei* are truly novel and without known homologs in the Rfam database. To further clarify the homologous distributions of all the detected sRNAs, BLASTN analysis was carried out across the 15 representative species of genus *Alcanivorax* with a threshold of ≥75% sequence identity and ≥75% of the sRNA’s length in the reference strain [39]. As a result, most of the sRNAs are highly specific to *A. dieselolei* and its close species, with more than 80% of sRNAs distributing in less than half of (≤7) the *Alcanivorax* species, especially in *A. dieselolei* and *A. xenomutans* (~60%) (Figure 5A and Table S14). Notably, a total of 41 sRNAs (7.5%) are broadly conserved among diverse *Alcanivorax* species with 75–100% sequence identities (Figure 5A,B). Interestingly, most of the broadly conserved sRNAs’ identities showed significantly positive correlations (Spearman-based analyses, *p* < 0.05 or 0.01) with their 16S rRNA-based phylogenies (Table S15), indicating the possibly vertical gene transfer of these conserved sRNAs. The inter-sRNAs showed a higher proportion in the broadly conserved sRNAs (12.2%, 5/41) than those in the whole sRNAs (6.2%, 34/549) (Figure 2 and Figure 5B). Except the Rfam-annotated core sRNAs, an antisense transcript of tmRNA (sRNA4307718) was also detected (Figure 5B), indicating an undetermined regulation role relevant to the tmRNA. In addition, an inter-sRNA (sRNA4758272) located between genes encoding ribosome biogenesis GTP-binding protein (B5T_04289) and Na+/H+ antiporter subunit G (B5T_04290) is also highly conserved in *Alcanivorax*, even with two homologous copies in some species (Figure 5B). In fact, this phenomenon of multiple copies was also found in some other broadly and poorly conserved sRNAs (Figure 5B and Table S14), suggesting that these sRNAs may play indispensable roles in their host *Alcanivorax* species. Notably, it does not exclude the existence of homolog even though a sRNA homolog is not detected here because the given threshold (≥75% identity and ≥75% coverage) may be overly conservative, particularly for the inter-sRNAs. Therefore, BLASTN analysis with a looser threshold (≥60% identity and ≥50% coverage) was further performed on the inter-sRNAs against all of the RefSeq_Representative_genomes of *Oceanospirillales* in the NCBI database. Similarly, most of the inter-sRNAs (27/34) are still *Alcanivorax*-specific, which is just distributed in several *Alcanivorax* species (Table S14). Only the four core sRNAs and the antisense transcript of tmRNA (sRNA4307718) are widely distributed across multiple genera of *Oceanospirillales*, of which 4.5S RNA and M1 RNA have homologs in 53 genera, followed by tmRNA (in 31 genera) and its antisense sRNA (32 genera), and 6S RNA (14 genera) (Table S14). Furthermore, it is noted that the sequence conservation of intra-sRNAs and asRNAs, especially those overlapped with CDSs, may be partly dependent on the conservation of their corresponding protein-coding genes. Homology also does not mean the real existence of sRNA due to the rapid evolution of sRNA in bacteria [28]; here, the sRNA homologs just provide some possibilities in *Alcanivorax*-related species.

### 2.9. Active Expressions of sRNAs Facilitating A. dieselolei to Adapt to Alkane Utilization

**Figure 6:**
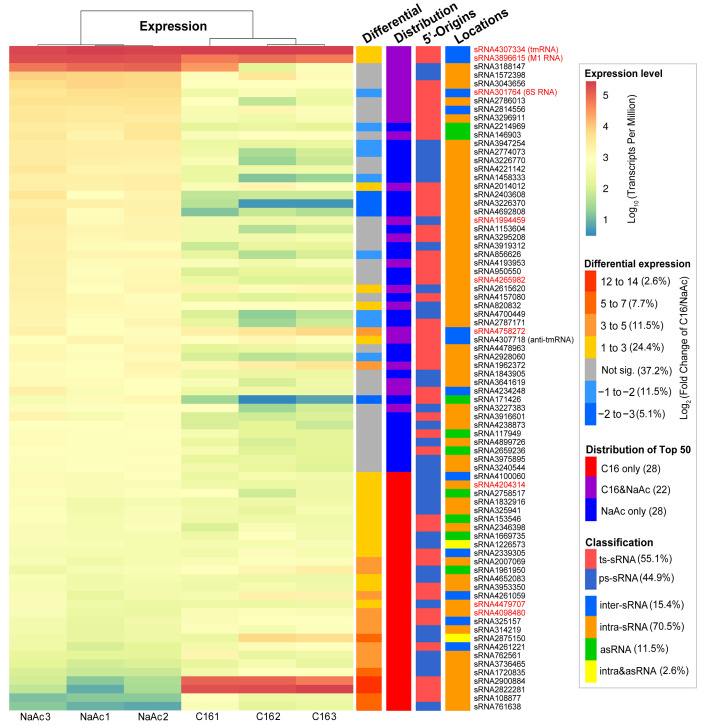
Expression of the top 50 sRNAs in alkane and non-alkane conditions. From left to right, the heatmap and colored bar charts show the expression levels in each sample of NaAc (non-alkane) or C16 (alkane) as carbon source, the differential expression of alkane vs. non-alkane conditions, the distribution of the top 50 highly expressed sRNAs in the two conditions and the classifications based on sRNA origins and locations. The sRNA names are listed behind, and the broadly conserved ones are in red.

Overall, the expression levels of the 549 sRNAs showed very broad ranges, spanning 6–7 orders of magnitudes under the alkane and non-alkane conditions (Table S16). The top 50 highly expressed sRNAs of each condition represented approximately 90–99% of the total number of hits in all samples (Table S16), and nearly half of them (22/50) are overlapped in both conditions with or without hexadecane as a growth substrate (Figure 6).

More than half (54.8%) of the 549 sRNAs showed similar expression levels, irrespective of alkane or acetate as the sole carbon and energy source, while 127 sRNAs (23.1%) were notably up-regulated and 121 (22.1%) were down-regulated in treatments of hexadecane compared to acetate (Figure S8). However, among the up-regulated ones, there are more sRNAs showing large expression fold changes (|Log2FC| > 3) (20.5%) than those (3.3%) in the down-regulated ones (Figure S8), indicating more intense expression responses of sRNAs during the alkane metabolism. Meanwhile, among the top 50 highly expressed lists of each condition, most of the sRNAs (36/50) were significantly up-regulated in alkane, but only a few sRNAs (13/50) were up-regulated in non-alkane treatments (Figure 6), which further supports the more active responses of sRNAs in alkane utilizing. Interestingly, there was a higher proportion of inter-sRNAs (12% vs. 4%) among the up-regulated sRNAs than the down-regulated ones in the alkane treatments (Figure S8), suggesting that more intergenic sRNAs participate in the alkane metabolism. In line with the above active sRNA responses to alkane, the key sRNA-chaperone Hfq was also up-regulated (~1.8 times) in alkane (Table S17), which can assist in the functioning of sRNAs during alkane utilization.

Among the actively responded sRNAs, there are three core sRNAs, i.e., tmRNA, M1 RNA and 6S RNA (Figure 6), which can be further deduced by their roles in alkane metabolic regulations based on their known conserved functions. All of the three core sRNAs are among the highly expressed sRNAs, in which tmRNA is the most abundant sRNA in either carbon source (Figure 6 and Table S16). The tmRNA and M1 RNA were significantly up-regulated while the 6S RNA was down-regulated in alkane treatments (Figure 6). Given the important roles of tmRNA in bacterial ribosome rescue and stress responses [60,72], and that bacterial alkane metabolism is usually associated with the membrane and oxidative stresses [73], we propose that the up-regulated tmRNA may be indirectly implicated in the global regulation of alkane metabolism through mitigating the stresses during the alkane degradation. The M1 RNA as the key component of RNase P that is a major player in the processing, maturation and decay of post-transcriptional RNA metabolism [44,58], and its up-regulation indicates the actively sRNAs-mediated RNA turnovers during alkane metabolism. Meanwhile, the 6S RNA is an important global repressor of transcription initiations [17,55], thus its down-regulation in alkane means more genes would increase their transcriptions. In line with this inference, more genes regardless of sRNAs or mRNAs are detected to significantly increase their expressions responding to the alkane compared to the control (Tables S16 and S17). In other words, *A. dieselolei* may globally switch the expression of related genes at the transcriptional level through the core 6S RNA to adapt the alkane utilization. Besides the 6S RNA riboregulator, other key protein-regulators may also participate in the process of transcriptional expression switching between distinct carbon sources. For instance, the H-NS (histone-like nucleoid structuring protein) DNA-binding protein (B5T_00005), as a major genome-wide transcriptional silencer [74], was also remarkably down-regulated in alkane (Table S17), suggesting the coordinated regulations of sRNA- and protein-regulators during alkane metabolism.

### 2.10. CsrA-Related sRNAs Likely Involved in Alkane Metabolism through Diverse Mechanisms

By far, the most well-known alkane metabolism regulatory mechanism mediated by sRNA is the multi-tier regulating strategies of the Crc/Hfq system in *Pseudomonas* [75]. However, there is no homologous protein of Crc in *Alcanivorax*. Another key global regulator CsrA (carbon storage regulator; also named Rsm, repressor of secondary metabolism) is widely distributed in this genus and shows a highly consistent synteny of ‘tRNA-CsrA-Aspartate kinase’ with neighboring genes (Table S12), implying its conserved function within *Alcanivorax*. CsrA mainly inhibits translation initiation through binding a conserved GGA motif in the 5′UTR of mRNA, and the inhibition can be relieved by CsrA-antagonized sRNAs (e.g., CsrB and CsrC) through molecular mimicry [76]. As a key global regulator, CsrA has been reported to regulate various physiological processes like carbon metabolism, iron metabolism, motility, cell envelope, secondary metabolism and biofilm formation [76,77,78,79], and the involvement of CsrA in bacterial alkane metabolism has never been reported before.

With the help of the CSRA_TARGET program [78], the potential targets of CsrA (B5T_03095) were first predicted in *A. dieselolei* (Table S18). Interestingly, the result indicated that some mRNAs of genes involved in the alkane-utilization-related pathways might be targeted by CsrA, such as *rubA* (B5T_04349) in the electron transfer during alkane hydroxylation, *aldH* (B5T_00039) and *exaA* (B5T_01640) in the aldehyde and alcohol oxidations, *proB* (B5T_03726) in the proline synthesis—which is the key component of the lipopeptide biosurfactant generated by *A. dieselolei* B-5 [80]—and the genes in the β-oxidation, such as *fadB* (B5T_01660) and *acdA* (B5T_04219) (Table S18). In addition, genes involved in iron metabolism, motility, central carbon metabolism and cell envelope were also in the targets list (Table S18), similar to previous reports in model bacteria *E. coli* and *B. subtilis* [76,77]. These results suggested that the global protein-regulator CsrA may directly participate in the alkane metabolic pathways.

**Figure 7:**
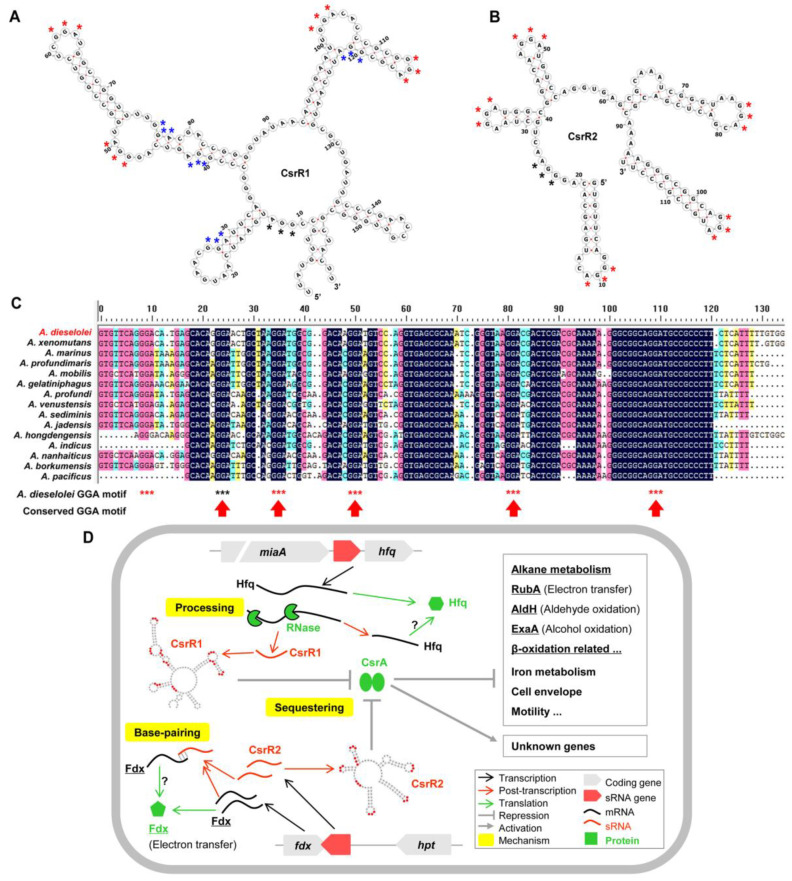
CsrA-related sRNAs and proposed regulation mechanisms in *A. dieselolei*. (**A**,**B**) show the secondary structures of the CsrR1 and CsrR2 conserved regions, respectively. (**C**) The alignments of CsrR2 homologous sequences in different species of *Alcanivorax*. The potential GGA motifs are marked using asterisks with different colors, red for motifs in loops, black for motifs in single-strand regions and blue for motifs at the junctions of the stem-loop. Secondary structures were calculated using RNAfold, and sequences were aligned with DNAMAN (version 8.0). (**D**) The proposed regulation mechanisms of the two CsrA-related sRNAs in alkane metabolism. The detailed notes are on the bottom right corner. Abbreviations: *miaA*, tRNA dimethylallyltransferase (B5T_00773); *hfq*/Hfq, Host factor (B5T_00774); RubA, Rubredoxin-NAD(+) reductase (B5T_04349); AldH, Aldehyde dehydrogenase (B5T_00039); ExaA, PQQ-dependent dehydrogenase (B5T_01640); *fdx*/Fdx, Ferredoxin (B5T_03135); *hpt*, Hpt domain-containing protein (B5T_03136). The mRNA/protein related to alkane metabolism is underlined. The question mark represents unknown effects.

Importantly, combined the InvenireSRNA prediction [81], Rfam alignment and secondary structure analysis, we identified two putative CsrA-related sRNAs (named CsrR1 and CsrR2) from the 549 sRNAs of *A. dieselolei* (Figure 4A and Figure 7A–C and Tables S10 and S18), which may sequester the CsrA regulator and therefore be indirectly involved in the alkane metabolism. CsrR1 originated from the 5′UTR of *hfq* (B5T_00774). Hfq is the most important chaperone interacting with sRNAs in most bacteria [49,50]. Our results indicate the intricate links among Hfq, CsrA and CsrA-related sRNA (Figure 7D). However, BLASTN-based analysis showed that homologs of CsrR1 only distribute in *A. dieselolei* and *A. xenomutans* (Figure 4B), implying that CsrR1-mediated regulatory relationships with CsrA and Hfq should be localized.

CsrR2 is independently transcribed through its own promoter—possibly recognized by sigma 70 (Table S5)—and the homologs are conserved in all *Alcanivorax* species (Figure 4B), indicating a housekeeping and conservative role in this genus. The syntenies of nearby genes are also highly consistent among *Alcanivorax* species (Figure 4C), with a same directional gene *hpt* encoded Hpt domain-containing protein and a divergent gene *fdx* encoded ferredoxin family protein (Table S12). Intriguingly, CsrR2 is also the antisense RNA of the neighboring *fdx* gene (Figure 4A,C), demonstrating that CsrR2 might be a dual-function sRNA through base-pairing and protein sequestering mechanisms (Figure 7D), reminiscent of some previous sRNAs (e.g., McaS and GadY) [82,83]. Given that the ferredoxin family protein (B5T_03135) is involved in the electron transfer of alkane hydroxylase [12], CsrR2 may add a regulatory tier to alkane degradation by direct antisense base-pairing with the corresponding mRNA (Figure 7D). Unexpectedly, the same genetic organization of CsrR2 and ferredoxin also occurs in another common alkane-degrader *Pseudomonas*, in which there is a highly conserved organization of *fdxA*-*rsmZ*, with *rsmZ* encoding the functional homologue of CsrR2 and *fdxA* encoding a ferredoxin [84]. The role of CsrA-related sRNAs is highlighted in alkane metabolism.

Based on their different secondary structures and expression patterns, the two CsrA-related sRNAs might play different roles in regulating alkane metabolism. CsrR1 contains five unpaired and four partially paired GGA motifs (Figure 7A), and CsrR2 has six unpaired GGA motifs (Figure 7B). Previous studies have shown that the affinity of CsrA-sRNA interaction is closely related to the secondary structure of sRNA [83,85], and therefore CsrR1 and CsrR2 should have distinct titration abilities to CsrA. Furthermore, the expression of 5′UTR-derived CsrR1 was dependent on the parental mRNA of *hfq*, and both were up-regulated in alkane, while CsrR2 was constantly expressed in both conditions (Tables S16 and S17). The distinctive responses to alkane indicate that CsrR2 likely plays a fundamental role in antagonizing CsrA, while the up-regulated CsrR1 may indirectly boost translations of the aforementioned alkane-metabolism-related genes through further sponging CsrA. Taken together, both CsrR1 and CsrR2 may affect alkane metabolism through diverse mechanisms, which are summarized in Figure 7D.

### 2.11. Candidate sRNAs Directly Related to the Key Genes of Alkane Metabolic Pathways

**Figure 8:**
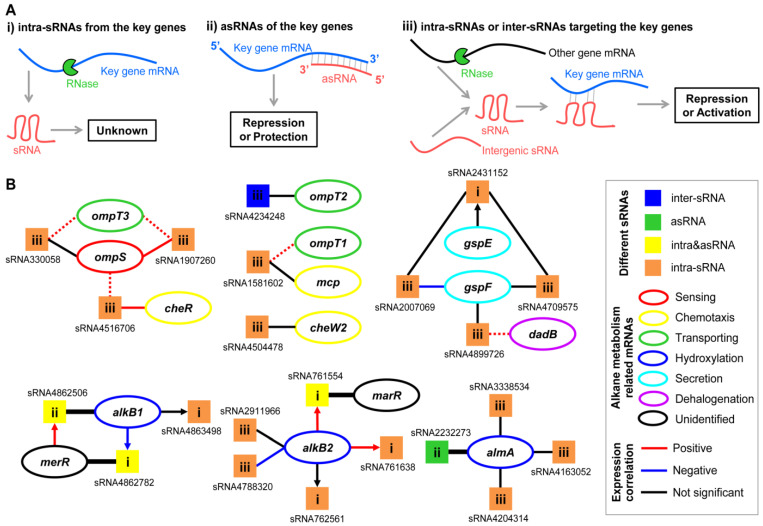
Putative relationships between the key genes in alkane metabolism and directly related sRNAs. (**A**) The three criteria to determine the directly related sRNAs with key genes of alkane metabolism. (**B**) Putative relationships between sRNAs and key genes. Each square represents a unique sRNA, and the sRNA identifiers are shown near the corresponding squares; the squares are colored according to the sRNA location-based classification. The ‘i, ii, iii’ in the squares correspond to the three criteria above. The ovals denote the mRNAs of key genes and are colored according to functions in alkane metabolism, and the related gene names are shown inside. The lines without an arrow show the matching relationships between sRNA and their targets, and the lines with arrows represent the sRNAs derived from the parental mRNAs. The *cis*-acting relationships (showing with wider lines) were deduced in terms of direct base-pairing for the asRNA and intra&asRNA, and the *trans*-acting relationships (showing with narrow lines) were inferred by IntaRNA prediction. The key genes in the top 10 most likely targets are shown with solid lines, and dotted lines show the potential targets beyond that range (top 20 to 100). The colors of lines or arrows indicate the correlationships of both of the connected ends based on Spearman correlation analyses of ssRNA-seq expression values in two carbon sources (n = 6).

In addition to the sRNAs mentioned above that may regulate alkane metabolism globally, more sRNAs that may directly participate in this regulatory process were also analyzed in *A. dieselolei* B-5. To this end, the previously characterized key genes of B-5 [6,7,12], including genes in processes of alkane sensing (*ompS*), chemotaxis (*mcp*, *cheW*, *cheR*), transporting (*ompT1–3*), hydroxylation (*alkB1*, *alkB2*, *ahpG*, *almA*), regulation (*almR*, *cyoD*) and other related processes (*fadB* in β-oxidation, *gspE* and *gspF* in putative secretion, *dadA* and *dadB* in haloalkane dehalogenation) were used to define candidate sRNAs directly related to the alkane metabolism key genesaccording to one of the following three criteria: (i) intra-sRNAs originating from the key genes, (ii) asRNAs of the key genes and (iii) intra-sRNAs or inter-sRNAs potentially targeting the key genes through the *trans*-acting mode based on predicted interactions (Figure 8A). To reduce the false-positive rate of target prediction, only the key genes in the top 10 ranking of IntaRNA results are shown here (Figure 8B).

As a result, we identified 21 candidate sRNAs from all 549 sRNAs, and most of the key genes in various processes of alkane metabolism were predicted to be targeted by these sRNAs through *cis*- and/or *trans*-acting mechanisms (Figure 8B and Table S19). For instance, for OmpS as the essential sensor and chemotaxis trigger of extracellular alkane in *A. dieselolei* [7], its mRNA is potentially targeted by three *trans*-acting intra-sRNAs, and these sRNAs also shown as interacting potentials with other mRNAs of key genes in alkane chemotaxis (*cheR*) and cross-membrane transport (*ompT3*), indicating that multiple processes of alkane metabolism might be closely linked and co-regulated by sRNAs. Moreover, other key genes in alkane transport (*ompT2* and *ompT1*) and chemotaxis (*mcp* and *cheW2*) are also predicted to be directly targeted by intergenic or intragenic *trans*-acting sRNAs (Figure 8B and Table S19). As a powerful alkane-degrader, *A. dieselolei* possesses multiple different alkane hydroxylases to deal with various alkanes [7,8,12]. Three of the identified hydroxylases, including AlkB1, AlkB2 and AlmA for initiating the oxidation of alkanes with different chain lengths and structures, are the parental mRNAs and/or direct targets of several sRNAs (Figure 8B). For the gene *alkB1*, the adjacent gene of the MerR family was supposed to be its transcriptional regulator [12,13]. Here, we found that two sRNAs were processed from the mRNAs’ 3′-ends of the above two genes and overlapped with the transcripts of *alkB1* and *merR*, indicating mutual *cis*-acting regulations of the two genes at the post-transcriptional level (Figure 8B). Expression of *alkB2* may be negatively regulated by two *trans*-acting intra-sRNAs (sRNA4788320 and sRNA2911966), and *alkB2* is also the parental mRNA of three sRNAs, in which sRNA761554 from the 3′UTR may antagonize the neighboring gene expression of a transcriptional regulator of the MarR family to indirectly influence the iron metabolism of *A. dieselolei*. With AlmA as the key hydroxylase of long-chain alkanes [7], multiple sRNAs showed possible *cis*- and *trans*-acting interactions with it (Figure 8B). In addition, the mRNA of *gspF*, which may affect alkane metabolism through secretion, shows potential interactions with three *trans*-acting intra-sRNAs, and sRNA2431152 processed from the 3′-end of *gspE* mRNA may sponge two of the above intra-sRNAs (Figure 8B and Table S19), suggesting sRNAs-mediated intricate regulations within an alkane metabolism-related operon (*gspE*-*gspF*). Among all the above 21 candidate sRNAs, there is only one broadly conserved sRNA (sRNA4204314) across the genus *Alcanivorax* (Figure 5 and Table S19), indicating that most of the directly related sRNAs in the alkane metabolic pathways are highly species-specific.

Therefore, sRNA may play a central role in alkane metabolism through diverse and flexible ways. The intricately mixed relationships among sRNA, mRNA and protein, like ‘one-to-many’ or ‘many-to-one’, collectively constitute the competitive alkane metabolic networks in *A. dieselolei*. On the basis of results in this study, the details of sRNA-mediated alkane metabolic regulations still remain to be determined relying on more experimental verifications (for example, with a GFP reporter system or an EMSA) in future.

## 3. Materials and Methods

### 3.1. Bacterial Strain, Growth Conditions and RNA Extraction

The *A. dieselolei* B-5 is a type strain of *Alcanivorax* isolated from the surface water of the Bohai Sea [8], and the strain is obtained from the Marine Culture Collection of China (MCCC1A00001^T^). It was cultivated in the artificial sea-water medium (ASM) using alkane *n*-hexadecane (0.5%, *v*/*v*) and non-alkane acetate (1.0%, *w*/*v*) as the sole carbon sources, respectively. The mid-log phase (OD600 ~ 1.0) bacterial cells were harvested at 4°C by centrifugation, then the pellets were lysed in 1 mL of TRIzol Reagent (Invitrogen, Waltham, MA, USA), and all samples were subsequently stored at −80 °C until RNA extraction. Three biological replicates were collected for each condition. The total RNA was extracted using a TRIzol reagent according to a previous description [86]. More details are listed in the Supplementary File.

### 3.2. Multiple Types of RNA-seq Libraries Preparation and Sequencing

Three types of RNA-seq libraries, including differential RNA-seq (dRNA-seq), fragmented strand-specific RNA-seq (ssRNA-seq) and ribosome profiling sequencing (Ribo-seq), were prepared to target the 5′-ends of transcripts with single-nucleotide resolution to determine the expression ranges and levels of transcripts, and to evaluate the coding potential of sRNA candidates, respectively.

The dRNA-seq libraries were prepared according to a previous description which could distinguish the primary and processed transcripts through a 5′P-dependent terminator exonuclease (TEX) treatment [33], and a procedure of RNA size (~50- to 500-nt) selection was added in the process to enrich the small RNAs. After that, the Illumina sequencing was performed on a HiSeq 4000 platform at the Cloud-Seq Biotech (Shanghai, China) according to the manufacturer’s instructions. The dUTP-based strand-specific ssRNA-seq libraries were constructed according to the previous protocols [87,88] at Majorbio Bio-Pharm Technology (Shanghai, China). Then, a HiSeq 4000 platform (Illumina, San Diego, CA, USA) was used for paired-end (2 × 150 bp) sequencing following the manufacturer’s instructions. The Ribo-seq libraries’ preparation and sequencing were performed according to previous studies [89,90] in Gene Denovo Biotechnology (Guangzhou, China). The cDNA libraries were sequenced using the Illumina HiSeq X10 sequencing system. More descriptions on the library construction are detailed in the Supplementary File.

### 3.3. Reads Mapping of Multiple RNA-seq Data

For all of the RNA-seq data, the sequencing reads were quality checked according to Illumina standards and then converted into FASTQ format. The READemption tool (version 2.0.1) was used to perform the read mapping with its subcommands of ‘create’ and ‘align’ against the reference genome of *A. dieselolei* B-5 [91,92]. When aligning with the genome, the parameters of ‘-Q 20′ and ‘-l 20′ were set to exclude the sequences with low sequencing qualities (Phred scores < 20) and shorter than 20-nt, and an extra parameter ‘-c’ was added in the read mapping of dRNA-seq to clip the polyA tails. For dRNA-seq, the single-end data that targeted the 5’ RNA ends were used for read mapping, while both-ends data of ssRNA-seq and Ribo-seq were used for the read mapping.

### 3.4. Visualization of Aligned Reads

To visualize the mapped results of dRNA-seq and corresponding ssRNA-seq, the READemption subcommand ‘coverage’ was used to generate strand specific coverage files based on the read alignments with default parameters. Finally, the data were visualized in the Integrative Genomics Viewer (IGV, version 2.3.68) [93], with the whole genome sequence (fasta format), the genomic annotation file (gff format) and the coverage files (wiggle format, normalized by the total number of aligned reads and multiplied by one million) as inputs.

### 3.5. Identification of TSSs, PSSs and Transcripts

The ANNOgesic pipeline (version 1.1.4) was used to predict and annotate numerous features through integrating a suite of tools based on the dRNA-seq and ssRNA-seq data [32], and all parameters were kept at the default values if not specified. The TSSs and PSSs were identified using the integrated TSSpredator tool in ANNOgesic (subcommand: ‘annogesic tss_ps’) with the programs of ‘TSS’ and ‘PS’, respectively, in which the relative enrichment of reads was compared between the TEX-treated and TEX-untreated samples [32,47]. The identified TSSs and PSSs were further improved by manual curation based on the visualized read coverage plots in IGV. The coverage-based transcript detection strategy in ANNOgesic (subcommand: ‘annogesic transcript’) was used to predict the transcripts through the input of ssRNA-seq data.

### 3.6. Determination of sRNA Candidates

To detect the sRNA candidates, we first used the ANNOgesic’s subcommand ‘annogesic srna’ to scan all the possible sRNAs (both intergenic and CDS-overlapped sRNAs), and added a parameter of ‘--detect_srna_in_cds’ with the inputs of the transcripts, coverage files, TSSs, PSSs and genome annotation. Meanwhile, the promoter table and terminator file generated by the subcommands of ‘annogesic promoter’ and ‘annogesic terminator’, together with the parameters of ‘--compute_sec_structures’ and ‘--srna_database_path’, were also included in the analyzing process to assist the filter of sRNAs. After that, each of the obtained sRNA candidates was manually verified based on the distributions, abundances and peak shapes of the mapped reads in the IGV visualization.

In addition, to overcome the potential bias of the ANNOgesic results due to the selection of parameters, we also manually inspected the distribution of all the mapped reads in IGV across the whole genome to complement the missed sRNA candidates. As a result, only the sRNA candidates that fit all the following criteria are included in the final sRNAs list: (i) sRNAs have mapped reads both in the dRNA-seq and ssRNA-seq results (to exclude the false-positive introduced by single strategy of library construction); (ii) the sRNAs’ abundances normalized by total reads number in ssRNA-seq are ≥10 in at least one carbon source condition (to exclude the very low abundance); (iii) the peak plots of sRNAs are with good shapes, i.e., there are TSSs or PSSs at the 5′-ends, and the 3′-ends are with terminators, PSSs or sharp coverage decreases (to lower the interference from degrading fragments and overlapped transcripts) [39,47].

### 3.7. Analyzing sRNA Primary Sequences and Secondary Structures

The CD-HIT was used to cluster the primary sequence similarity of sRNAs, with thresholds of over 70% sequence identity and 90% sequence length (parameters: -c 0.7, -aS 0.9 and default for others) [94]. The lengths and G + C contents of sRNAs, CDSs, rRNAs and tRNAs were calculated by BioEdit (version 7.2.5) according to their sequences [95]. The minimum free energy (MFE) of RNAs was calculated using RNAfold integrated in the RNA Workbench 2.0 with the default settings (Galaxy Version 2.2.10.4, https://rna.usegalaxy.eu/, accessed on 12 March 2022) [96,97]. To remove the bias from the sequence length, the normalized MFE (NMFE) was calculated by dividing the RNA sizes [21,26]. PlotsOfData was used to draw boxplots of the above features [98], and their distributions were compared using a Wilcoxon rank sum test calculated by a ‘PMCMRplus’ package in R. The RNAfold web server (http://rna.tbi.univie.ac.at//cgi-bin/RNAWebSuite/RNAfold.cgi, accessed on 13 May 2022) was used to predict and visualize the centroid secondary structures of sRNAs with the default parameters [97].

### 3.8. Motif Analyzing the Sequences Neighboring TSSs and PSSs

The putative promoter motifs were detected by scanning the 50-nt upstream of TSSs using MEME (https://meme-suite.org/meme/tools/meme, accessed on 13 July 2020) [32,99]. The upstream 100-nt sequences of TSSs were further analyzed by the iPromoter-2L, which can provide a high predictive power for different types of bacterial promoters [42]. The consensus motif of the sequences that spanned up- and down-stream 10-nt of the PSSs was aligned and visualized with WebLogo 3 (http://weblogo.threeplusone.com, accessed on 25 February 2022). The above sequence extraction is carried out using a customed Perl script.

### 3.9. Coding Potential Evaluation of sRNAs

Considering most of the intra-sRNAs should have coding potential in the same frame as the corresponding CDSs, they were therefore excluded from this analysis. The open reading frames (ORFs) in the asRNAs (n = 113) and inter-sRNAs (n = 34) were predicted using the ORF Finder (www.bioinformatics.org/sms2/orf_find.html, accessed on 4 December 2022). Only the ORFs, with lengths ≥ 20 aa and with complete start (AUG) and stop codons (UAA, UAG, UGA), were retained as potential sORFs. Moreover, the filtered high quality reads of Ribo-seq were mapped to the detected sRNAs using Bowtie2 [100], and the mapped sRNAs were further used to evaluate their coding potential by the ORF Finder. Blastp was used for homology searches of the potential sORFs against the non-redundant protein sequences (nr) [101].

### 3.10. sRNA Annotation and Homologs Analysis

All the sRNA candidates were aligned and annotated within the Rfam database (version 14.8, https://rfam.org/search/batch, accessed on 11 May 2022). The 15 reference genomes of *Alcanivorax* were downloaded from NCBI Datasets (https://www.ncbi.nlm.nih.gov/datasets/genomes, accessed on 17 May 2022). Then, BLASTN was used for similarity searches of the Rfam-annotated sRNAs against the reference genomes with relative loose parameters (-perc_identity 50 -qcov_hsp_perc 50 -evalue 0.00001) for the identity and coverage [102]. The gene synteny and Rfam annotation of the obtained sRNA homologs were performed to further verify their conservation. For the gene synteny analysis, the locations of sRNA neighboring genes are determined by the BLASTN result of hsp (high-scoring segment pair) hit regions, and the gene annotations are based on the Prokaryotic Genome Annotation Pipeline (PGAP) [103]. To reduce the false positives, except the Rfam-annotated sRNAs, we adopted more stringent BLASTN parameters (-perc_identity 75 -qcov_hsp_perc 75 -evalue 0.00001) to search the homologs of the remaining sRNAs in *Alcanivorax* according to a previously described threshold [39].

### 3.11. Expression Analysis of sRNAs

We used the ‘gene_quanti’ and ‘deseq’ subcommands of READemption to calculate the reads number overlapping the identified sRNAs and the differential expression in alkane and non-alkane conditions with the default parameters [91,104]. The expression level and change are represented by TPM (Transcripts per Million) normalized read counts and Log2FoldChange with an adjusted *p* value. Differentially expressed genes were defined as the adjusted *p* value < 0.05 and the absolute values of TPM Log2FoldChange ≥ 1 between the two conditions. The heatmap and volcano plots were used to visualize the gene expression levels and changes via the Heatmapper [105] and VolcaNoseR [106], respectively.

### 3.12. Predictions of CsrA-Related sRNAs and CsrA Targets

CsrA-related sRNAs were identified with the assistance of the R package “InvenireSRNA”, which integrated sequence- and structure-based features to train machine-learning models to detect the bacterial sRNAs in the CsrA pathway [81]. All the sRNA candidates and the extracted intergenic sequences of the whole genome were scanned using the InvenireSRNA to discover all the possible CsrA-related sRNAs in *A. dieselolei*. The putative CsrA-repressed targets across the whole genome were identified using the predictive algorithm CSRA_TARGET [78], which is based on the sequence feature of the conservative CsrA binding motif (ANGGA) in regions around the Shine–Dalgarno (SD) sequence.

### 3.13. Targets Prediction of sRNAs

The IntaRNA 2.0 program, which enables fast and accurate prediction of RNA–RNA interactions by integrating seed constraints and interaction site accessibility [107], was used to predict the mRNA targets of the trans-acting sRNAs in the B-5 genome. With the inputs of sRNA sequences and B-5 genomic information (RefSeq ID: NC_018691), the ‘whole genome mode’ of IntaRNA was selected to target the potential regions of annotated genes using the default parameters (http://rna.informatik.uni-freiburg.de/IntaRNA/Input.jsp, accessed on 19 July 2021).

## 4. Conclusions

**Figure 9:**
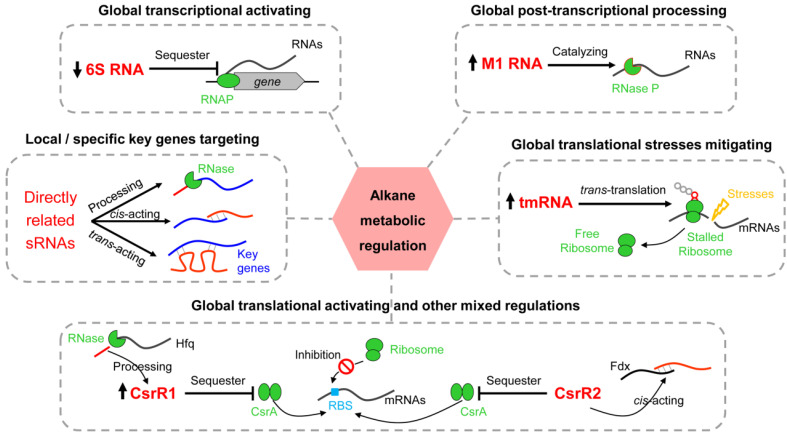
Hypothesized sRNA-mediated alkane metabolic regulations in *A. dieselolei* B-5. The key related sRNAs (red and largest font) and their potential regulating mechanisms are shown in each dotted box. The significant expression changed sRNAs responding to the alkane, which are marked with up and down arrows before them, representing up- and down-regulated expressions, respectively. The possible regulating effects of the sRNAs are indicated above each dotted box (black and bold font). Abbreviations: RNAP, RNA polymerase; Hfq, Host factor; Fdx, Ferredoxin; RBS, ribosome binding site.

Here, combining the advantages of dRNA-seq and RNA size selection strategy, we comprehensively captured the high-resolution sRNAs landscape in the marine-alkane-degrading bacterium *A. dieselolei* B-5. These sRNAs can originate from nearly everywhere in the genome through promoter-driven transcription or post-transcriptional RNA processing. RNase E likely plays a key role in the processing and maturation of sRNAs. Most of the sRNAs have no sORF-coding potential and are species-specific distributed in *Alcanivorax*. Some core sRNAs, including 6S RNA, M1 RNA and tmRNA, are first revealed as responding to alkane, which can likely reprogram the global gene expressions at multiple levels of transcription, post-transcription and translation to benefit the alkane utilization. Two novel CsrA-related sRNAs may also complementarily regulate alkane metabolism through sponging the global translation repressor CsrA, in concert with other mechanisms. Species-specific sRNA-mediated regulations may be widely involved in key processes of alkane sensing, chemotaxis, transporting and hydroxylation. Altogether, both core and local/specific sRNAs may collaboratively reshape the gene expressions at various levels through diverse mechanisms to optimize the alkane utilization in B-5 (Summarized in Figure 9), among which several core sRNAs maybe respond to the alkane and regulate gene expressions globally, and multiple specific sRNAs may concurrently target the key pathways in alkane metabolism. Our study opens up an important avenue for exploring sRNA-mediated regulatory networks and will stimulate further work in the identification of new functional sRNAs and novel regulatory mechanisms in alkane metabolism.

## Data Availability

The raw data have been deposited in the Genome Sequence Archive [108] in the National Genomics Data Center (NGDC, https://ngdc.cncb.ac.cn/gsa, accessed on 27 October 2022) under the accession numbers of CRA008650 (ssRNA-seq), CRA008652 (dRNA-seq) and CRA008653 (Ribo-seq), respectively. Other available data are uploaded to the figshare, including the data of IGV visualization, the ANNOgesic outputs and the results of IntaRNA-based targets prediction with the links of https://doi.org/10.6084/m9.figshare.18094298, https://doi.org/10.6084/m9.figshare.21159580 and https://doi.org/10.6084/m9.figshare.21195049, (All accessed on 31 October 2022) respectively.

## Supplementary Materials

The following supporting information can be downloaded at: https://www.mdpi.com/article/10.3390/ijms232415995/s1, Figures S1–S8: Supplementary figures.pdf; Tables S1–S19: Supplementary tables.xlsx; Supplementary file: Supplementary file.docx.
