# Supplementary material for: High-Resolution Small RNAs Landscape Provides Insights into Alkane Adaptation in the Marine Alkane-Degrader Alcanivorax dieselolei B-5
Source: Int J Mol Sci. 2022 Dec 15;23(24):15995. doi: 10.3390/ijms232415995 (PMC9788540; doi:10.3390/ijms232415995)
Supplement: Supplementary file 1 [file ijms-23-15995-s001.zip › ijms-2057672-Supplementary figures.pdf]

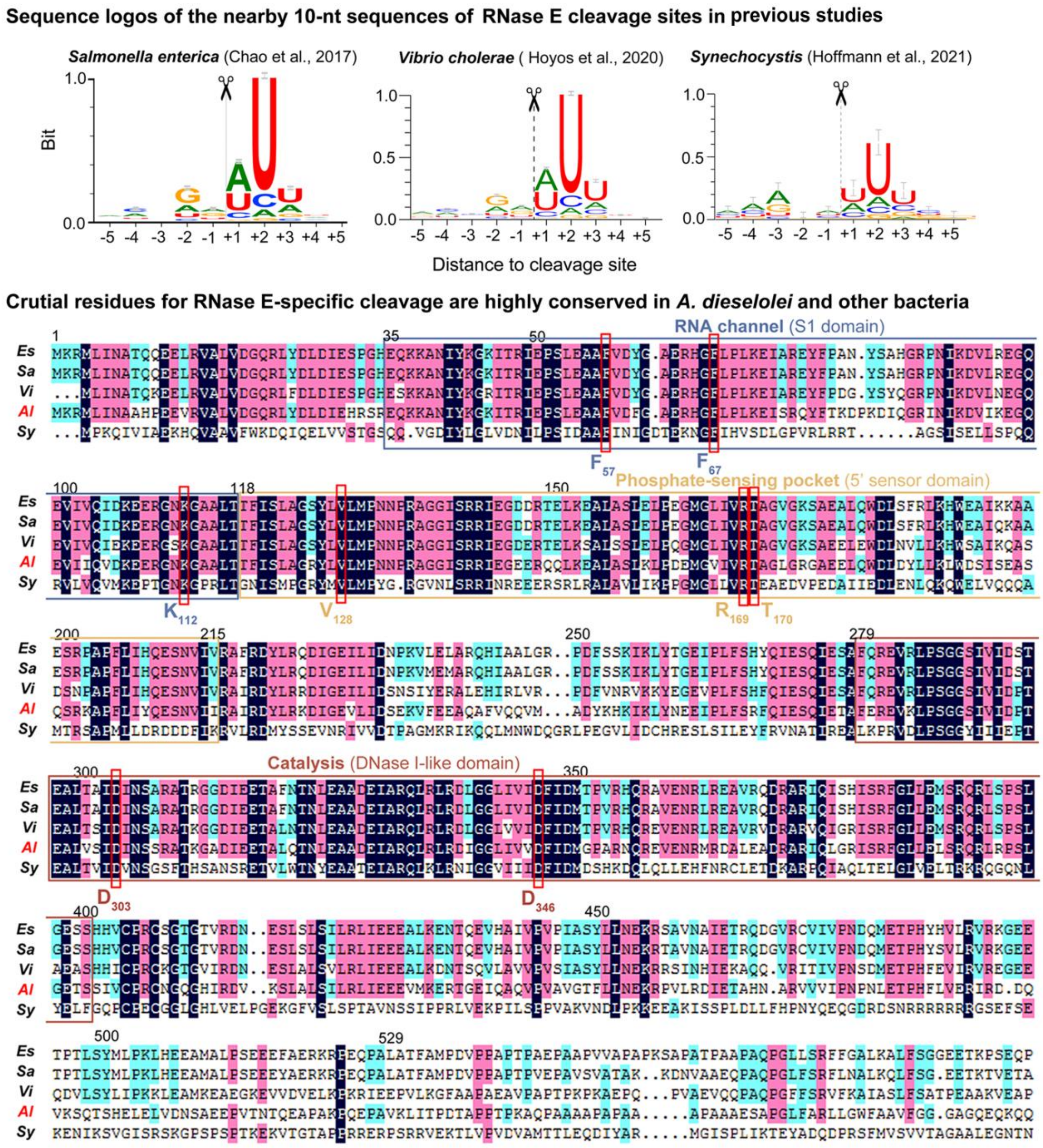

Figure S1 Sequence logos of the TSSs and PSSs, and key residues in RNase E of different species

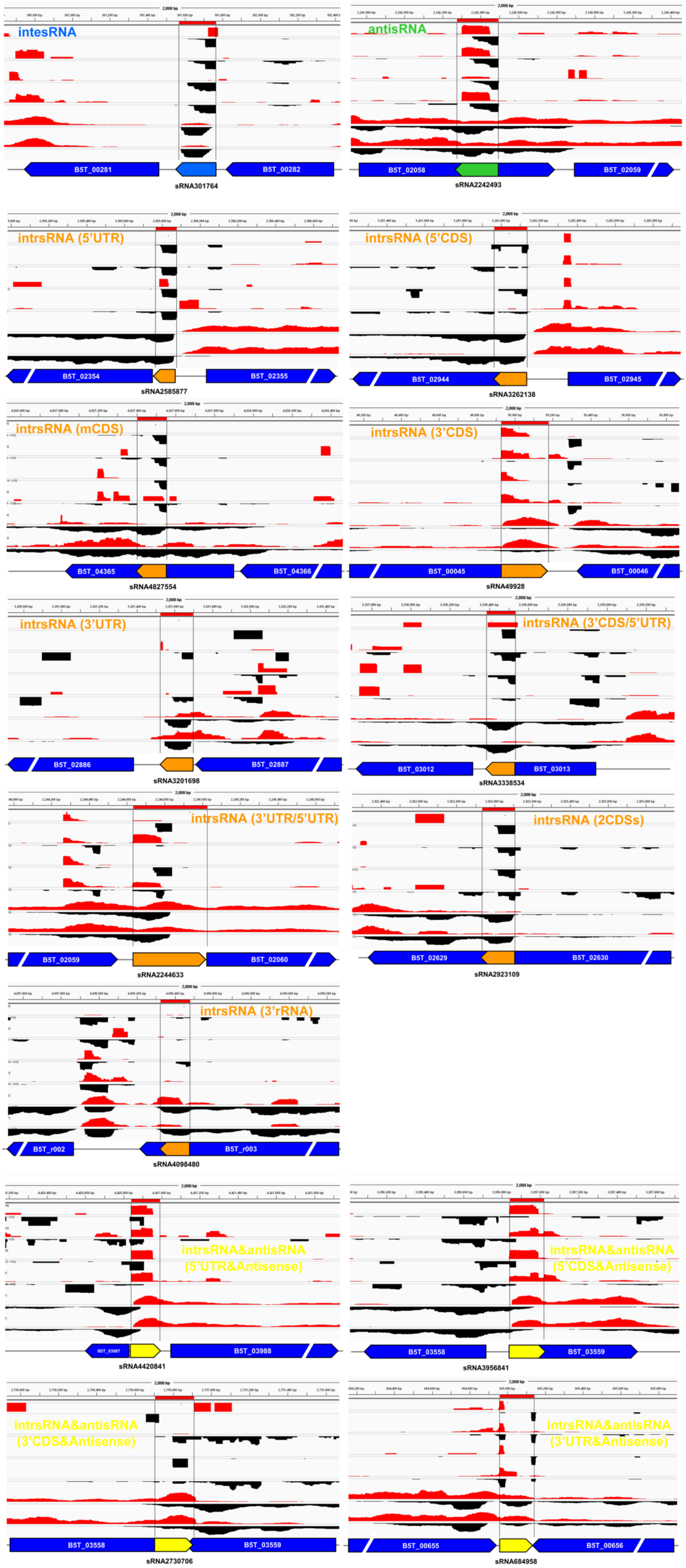

Figure S2 Examples of peak plots of different sRNA classifications based on the genomic locations

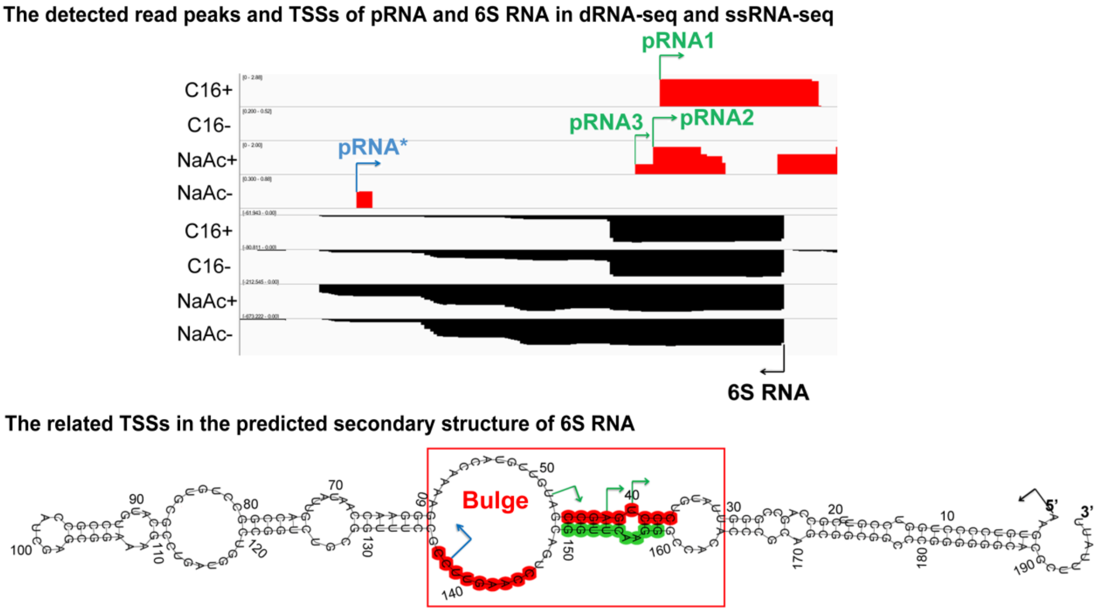

Figure S3 Putative pRNAs of 6S RNA in *A. dieselolei*

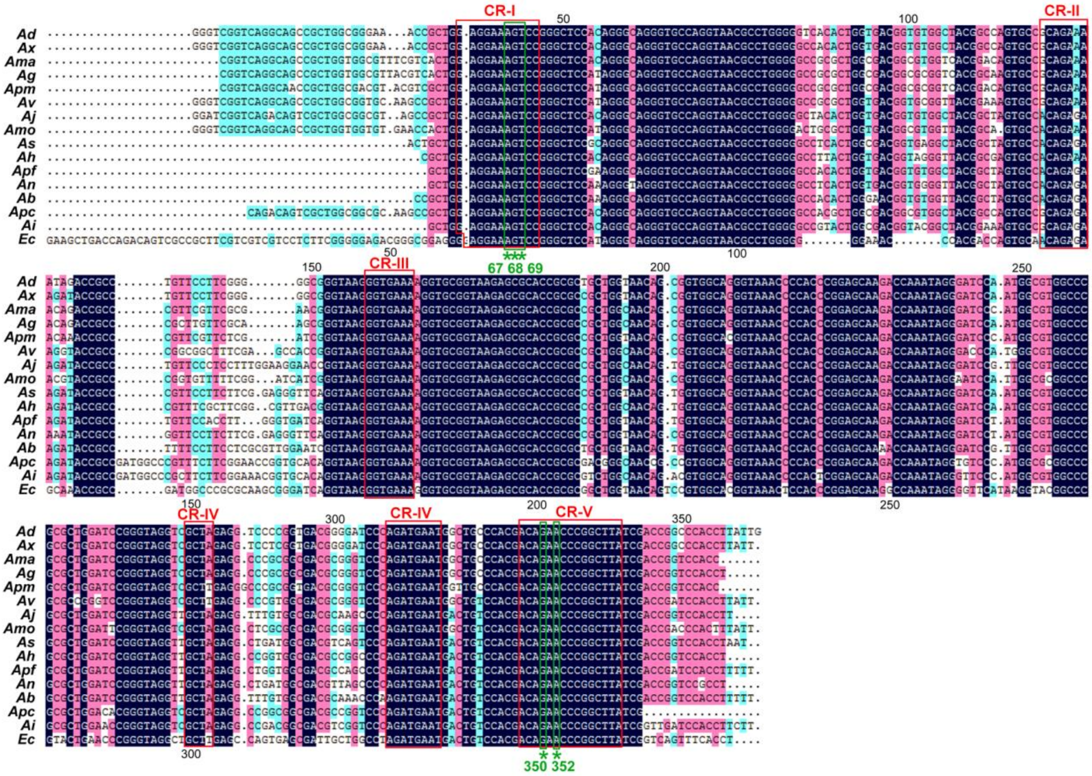

Figure S4 Conserved regions (CR-I to CR-V) and active sites (*) of M1 RNAs in different *Alcanivorax* species

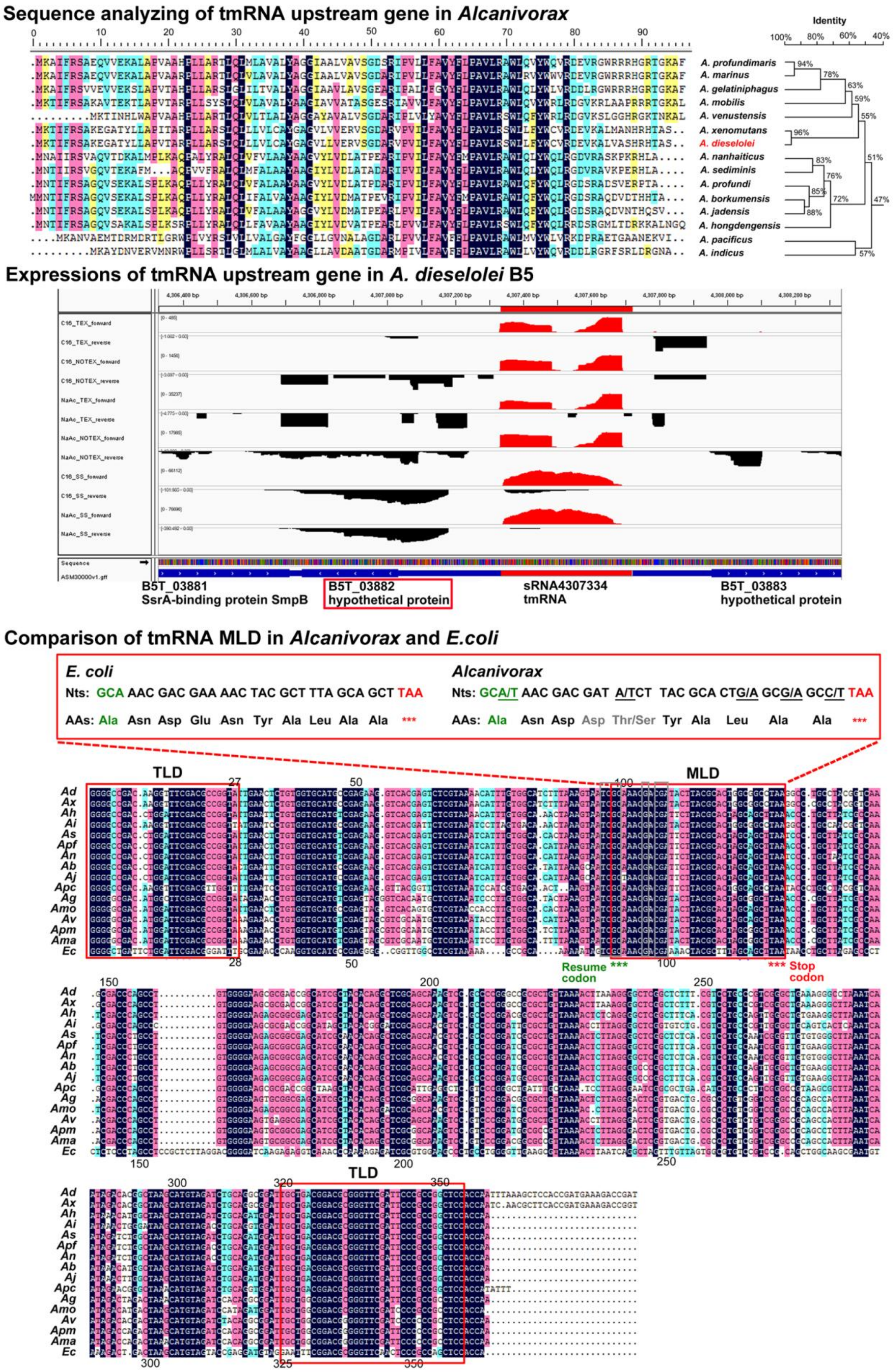

Figure S5 tmRNA upstream gene and MLD features in different *Alcanivorax* species

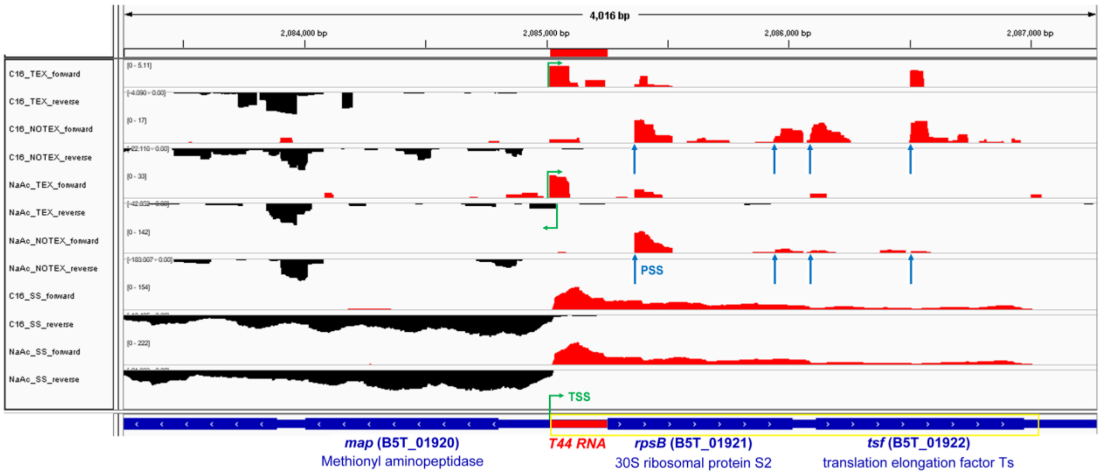

Figure S6 Expression peaks of T44 RNA and nearby genes

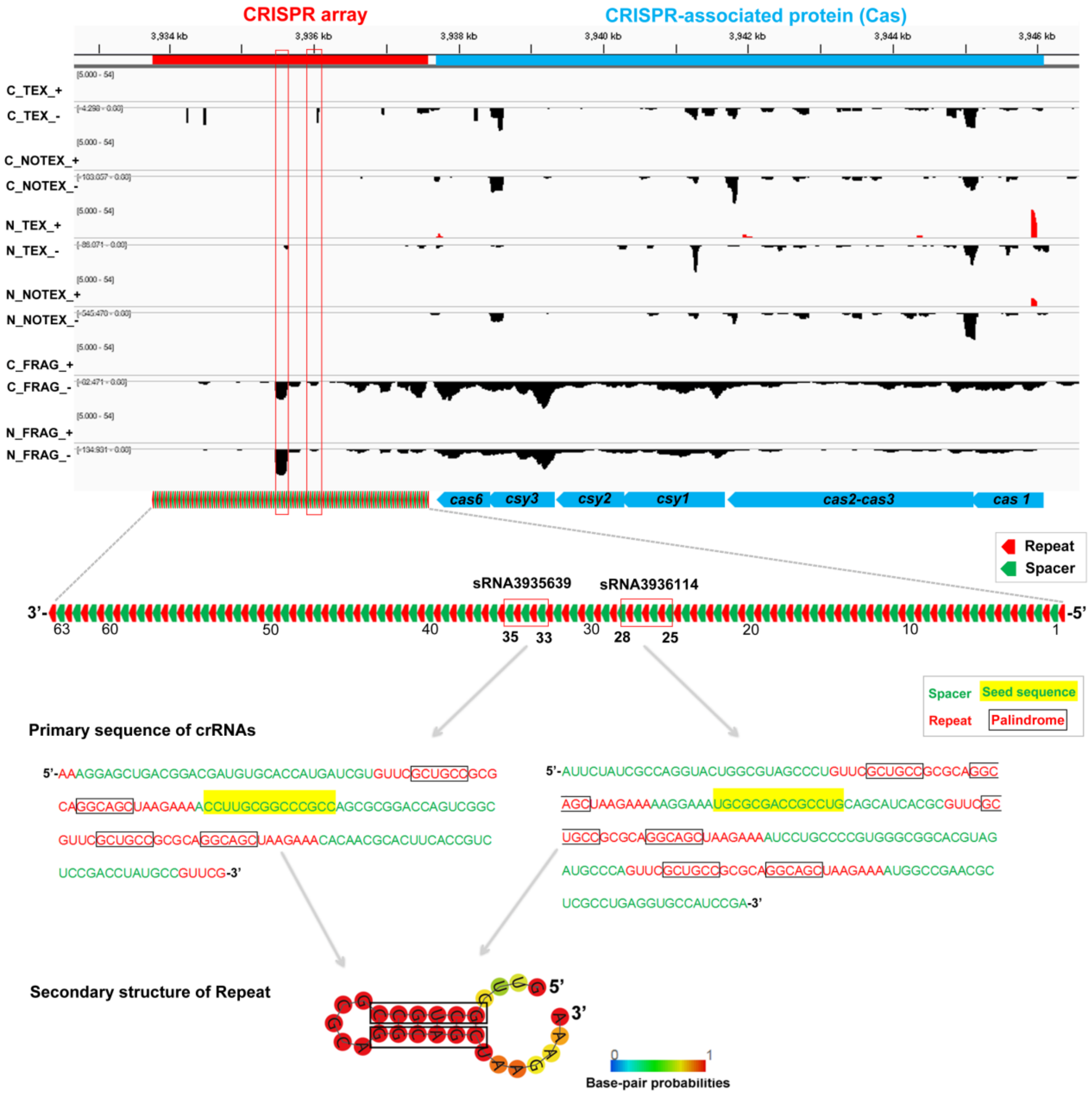

Figure S7 Expression and identificaion of CRISPR-Cas system in *A. dieselolei*

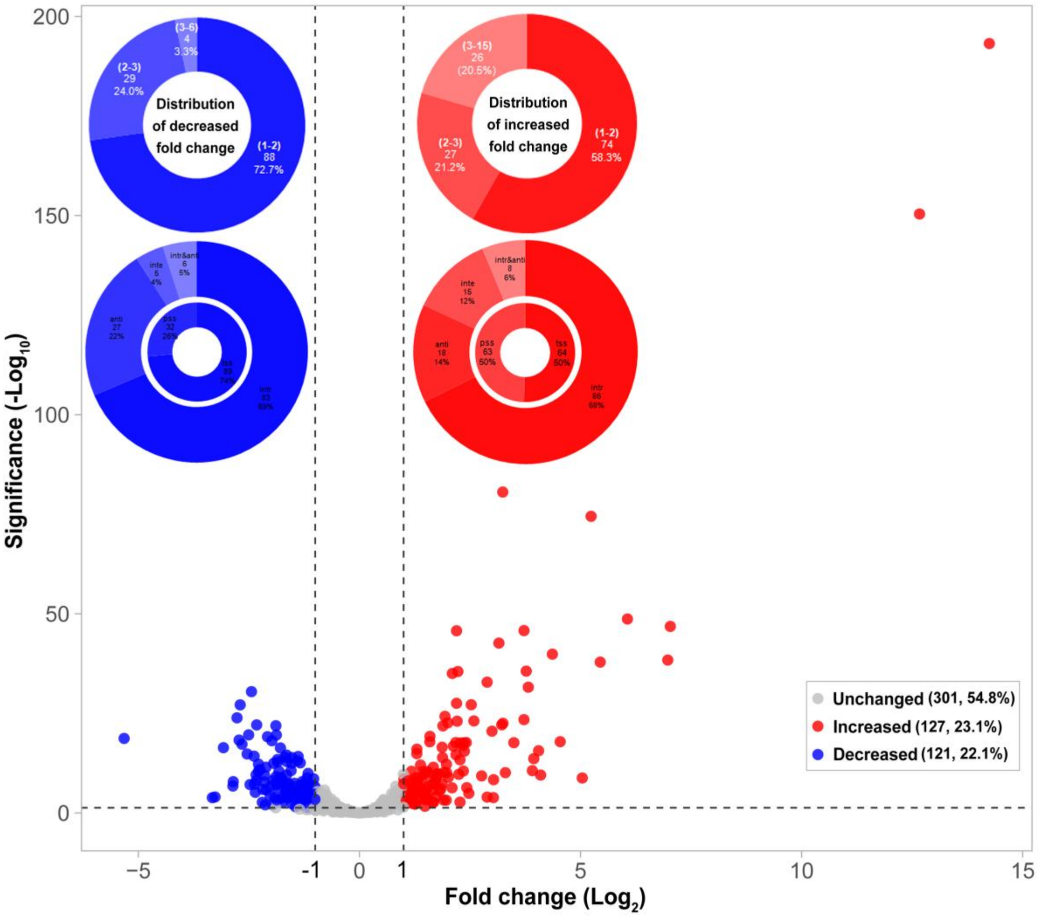

Figure S8 Volcano plots showing the differential expression in alkane versus acetate conditions
