# Supplementary material for: High-Resolution Small RNAs Landscape Provides Insights into Alkane Adaptation in the Marine Alkane-Degrader Alcanivorax dieselolei B-5
Source: Int J Mol Sci. 2022 Dec 15;23(24):15995. doi: 10.3390/ijms232415995 (PMC9788540; doi:10.3390/ijms232415995)
Supplement: Supplementary file 1 [file ijms-23-15995-s001.zip › ijms-2057672-supplementary.pdf]

Supplementary Materials and Methods

Bacterial strain cultivation and RNA extraction

Cultivation. The ASM was prepared as described previously (Liu and Shao, 2005), amended with lower pH (7.2) and less $MgSO_4·7H_2O$ (3.5 g/L) to avoid medium precipitation. All cultures were incubated at 28°C and spun at 180 r.p.m. for growth growth monitoring.

RNA extraction. Briefly, cell pellets corresponding to a total amount of ~3.0 OD_{600} were lysed using 1 mL of TRIzol, followed by adding 200 μL of chloroform and shaking vigorously. After centrifugation at 4°C, 12,000 g for 15 min, the supernatant was fully mixed with 500 μL of isopropanol. After another round of centrifugation, the pelle was washed twice using 70% ethanol, then air-dried and dissolved in RNase-free water. RNA purity and integrity were assessed using a Nanodrop 2000 Spectrophotometer (Thermo Scientific) and the Agilent Bioanalyzer 2100 system (Agilent Technologies), respectively.

Multiple RNA-seq libraries preparation and sequencing

dRNA-seq. Briefly, the residual genomic DNA and ribosomal RNAs were first removed by DNase I (NEB) and Ribo-Zero™ rRNA Removal Kit (Bacteria) (Epicentre) according to the manufacturers' instructions. Then, the RNA samples were poly(A)-tailed using *E. coli* poly(A) polymerase (NEB), and terminator™ 5'-phosphate-dependent exonuclease (TEX) (Epicentre) was used to degrade the processed transcripts with 5'-P structure and enrich the primary transcripts with 5'-PPP as previously described (Bischler et al. 2015). For each carbon source

condition, two treatments, with TEX (TEX+) and without TEX (TEX-), were carried out after poly(A)-tailing. Finally, all samples were treated with TAP (tobacco acid pyrophosphatase, Epicentre) to transform the 5'-PPP to 5'-P, then the Illumina sequencing RNA adapter was ligated to the 5'-P of TAP-treated RNAs using T4 RNA ligase (NEB). To obtain majority of the sRNAs with accurate 5'-ends, RNAs within 50-500 nt were size-selected using 8% polyacrylamide gel electrophoresis, and no fragmented step was performed before the cDNA library preparation (Bischler et al. 2015; Leonard et al., 2019). The cDNA libraries were constructed as previously described (Bischler et al. 2015), and then the Illumina sequencing was performed on a HiSeq 4000 platform at the Cloud-Seq Biotech (Shanghai, China) according to the manufacturer's instructions.

ssRNA-seq. Briefly, after removal of the residual genomic DNA and ribosomal RNAs as described for the dRNA-seq library, dUTP-based strand-specific libraries were created using the TruSeq™ Stranded Total RNA Library Prep Kit (Illumina) according to the manufacturer's protocols (Parkhomchuk, et al. 2009; Levin et al., 2010).

Ribo-seq. Briefly, chloramphenicol with a final concentration of 100 μg/mL was used for ribosome stalling, and cells were collected by centrifugation and immediately flash frozen in liquid nitrogen together with 400 μL bacterial lysis buffer as described previously (Wang et al., 2015). Then the ribosome footprints (RFs) were recovered according to previous studies (Ingolia et al., 2012; Wang et al., 2015). After obtaining the RFs, the Ribo-seq cDNA libraries were constructed using the NEBNext® Multiple Small RNA Library Prep Set for Illumina® (New England Biolabs, MA, USA)

according to the manufacturer’s instructions.

Experimental objectives and sampling strategies in this study

**Objective**	**Library type**	**Sampling strategy**	**Culture condition**
To accurately identify sRNAs with different origins and locations	dRNA-seq+ ssRNA-seq	Three independent biological replicates with similar OD_{600} values of the mid-log phase were merged into one mixture after RNA extractions for each condition	*n*-hexadecane vs. acetate as the only carbon source
To determine the expression boundaries and levels of sRNAs	ssRNA-seq	Three independent biological replicates with similar OD_{600} values of the mid-log phase for each condition	*n*-hexadecane vs. acetate as the only carbon source
To evaluate the coding potential of sRNAs	Ribo-seq	Two independent biological replicates with similar OD_{600} values of the mid-log phase for each condition	*n*-hexadecane vs. acetate as the only carbon source

Reference genomes information of the 15 *Alcanivorax* species used in this study

**Species name**	**Genome size (Mb)**	**G+C content (%)**	**Gene number**	**Genome level**	**Rference**
A. dieselolei B-5	4.928	61.5	4468	Complete	Lai et al. 2012, doi: 10.1128/JB.01813-12.
A. xenomutans JC109	4.35	61.5	3962	Scaffold	https://www.ncbi.nlm.nih.gov/data-hub/genome/GCF_900217905.1/
A. marinus SY10-13	4.175	65	3883	Contig	https://www.ncbi.nlm.nih.gov/data-hub/genome/GCF_016785095.1/
A. gelatiniphagus MEBiC 08158	4.215	65.2	3887	Contig	https://www.ncbi.nlm.nih.gov/data-hub/genome/GCF_005938655.1/
A. profundimaris ST75FaO-1	4.036	66	3777	Scaffold	https://www.ncbi.nlm.nih.gov/data-hub/genome/GCF_015265435.1/
A. mobilis MT13131	4.1	63	3782	Contig	https://www.ncbi.nlm.nih.gov/data-hub/genome/GCF_002864685.1/
A. venustensis ISO4	3.544	64.5	3393	Contig	https://www.ncbi.nlm.nih.gov/data-hub/genome/GCF_015356855.1/
A. jadensis T9	3.629	58	3327	Contig	https://www.ncbi.nlm.nih.gov/data-hub/genome/GCF_000756655.1/
A. sediminis PA15-N-34	3.798	57	3451	Contig	https://www.ncbi.nlm.nih.gov/data-hub/genome/GCF_009601165.1/
A. nanhaiticus 19-m-6	4.133	56	3806	Contig	https://www.ncbi.nlm.nih.gov/data-hub/genome/GCF_000756665.1/
A. profundi MTEO17	3.737	57	3506	Contig	https://www.ncbi.nlm.nih.gov/data-hub/genome/GCF_003597125.1/
A. hongdengensis A-11-3	3.665	60.5	3498	Contig	Lai et al. 2012, doi: 10.1128/JB.01849-12.
A. borkumensis SK2	3.12	54.5	2826	Complete	Schneiker et al. 2006, doi: 10.1038/nbt1232.
A. indicus SW127	3.445	62.5	3161	Contig	https://www.ncbi.nlm.nih.gov/data-hub/genome/GCF_003259185.1/
A. pacificus W11-5	4.168	62.5	3785	Complete	Lai and Shao 2012, doi: 10.1128/JB.01845-12.

The data were collected from the NCBI Datasets before the July, 2021.
